# Reframing clathrin-mediated viral entry: evidence standards, context dependence and host-directed vulnerabilities

**DOI:** 10.3389/fcimb.2026.1929024

**Published:** 2026-10-05

**Authors:** Qinying Wang, Liran Xu, Yangang Sun, Yuting Xia, Jingyu Yue

**Affiliations:** 1 Department of The First Clinical Medical College, Henan University of Chinese Medicine, Zhengzhou, China; 2 Department of AIDS Clinical Research Center, The First Affiliated Hospital of Henan University of Chinese Medicine, Zhengzhou, China; 3 School of Medicine, Henan University of Chinese Medicine, Zhengzhou, China

**Keywords:** AP-2, clathrin-coated pit, clathrin-mediated endocytosis, dynamin, endosomal entry, evidence hierarchy, single-particle tracking, viral entry

## Abstract

Viral entry is the first obligatory step of productive infection and a rational point for host-directed antiviral intervention. This structured narrative review synthesizes evidence from database searches and citation tracking performed up to 18 June 2026. Clathrin-mediated endocytosis (CME) can internalize virus-receptor complexes and deliver them to endosomal compartments that support penetration, fusion, capsid remodeling, uncoating or genome release. However, CME should be interpreted as a series of experimentally separable checkpoints rather than as a single pathway label. Viral use of CME varies with strain, receptor and cofactor availability, host-cell type, membrane composition, protease environment, inoculum size and synchronization conditions. Strict CME-dependent entry is assigned only when direct perturbation of core or proximal CME machinery, supported by rescue or an orthogonal approach, converges with stage-resolved evidence of productive virion progression through clathrin/AP-2-positive coated pits or vesicles and an early functional entry readout in a defined virus-receptor-cell context. Pharmacological findings are treated as supportive rather than universally mandatory when stronger direct mechanistic evidence is available. The review integrates receptor organization, cargo-specific adaptor recruitment, membrane lipids, particle geometry, actin dynamics and virus-induced remodeling with a transparent evidence framework that distinguishes CME-associated, CME-involved and CME-dependent entry. It further separates mechanistic probes from host-directed strategies targeting membrane sphingolipids, TPC2-dependent endolysosomal trafficking, NPC1/cholesterol homeostasis, PIKfyve and kinase-regulated trafficking. Translational priorities are framed as context-dependent opportunities requiring target engagement, disease-relevant models and *in vivo* validation rather than as validated consequences of global CME blockade.

## 1 Introduction

Viral entry is the process by which receptor engagement is converted into membrane penetration, capsid remodeling, uncoating or genome release. Because these early events shape infection efficiency, tissue tropism and host range, entry pathways remain attractive but methodologically demanding targets for host-directed antiviral research (Smith and Helenius, 2004; Mercer et al., 2010; Kalia and Jameel, 2011).

Among known entry routes, CME has been extensively characterized in classical viral-entry models, including Semliki Forest virus, influenza A virus, vesicular stomatitis virus, dengue virus, minor-group rhinovirus and JC polyomavirus (Helenius et al., 1980; Snyers et al., 2003; Rust et al., 2004; van der Schaar et al., 2008; Cureton et al., 2009; Mayberry et al., 2019). In viral-entry studies, however, CME should be treated as a sequence of checkpoints rather than as a binary label. Virus-receptor complexes may be recruited to clathrin-coated pits (CCPs), internalized through dynamin-dependent scission and delivered to endosomal compartments that support acidification, protease activation, fusion, penetration or genome release (Ehrlich et al., 2004; Rust et al., 2004; Chen and Zhuang, 2008; van der Schaar et al., 2008; Cureton et al., 2009; Mercer et al., 2010). A decrease in endpoint infection after perturbing one of these processes does not automatically identify the affected step.

A central challenge is pathway plasticity. Entry-route selection can vary with viral strain or particle state, receptor and cofactor abundance, host-cell type, membrane tension, lipid composition, protease availability, inoculum size and synchronization conditions (Barrow et al., 2013; Cossart and Helenius, 2014; Rennick et al., 2021). The same virus may therefore use CME, macropinocytosis, lipid-raft/caveolae-associated uptake, clathrin-independent dynamin-dependent uptake or direct plasma-membrane fusion in different experimental systems (Mercer et al., 2010; Kalia and Jameel, 2011; Barrow et al., 2013; Cossart and Helenius, 2014). For this reason, “CME-dependent” should not be used without specifying the virus, receptor context, host-cell model and assay window.

This review uses a stage-resolved evidence framework to interpret CME-related viral-entry studies. First, it summarizes CME stages that are most relevant to viral internalization and endosomal delivery. Second, it defines operational evidence categories—CME-associated, CME-involved and CME-dependent entry—and applies them to representative human-relevant and model viruses. Third, it evaluates pharmacological tools and host-directed modulators used to interrogate CME, dynamin-dependent scission and CME-associated endosomal entry (Gillespie et al., 2013; Elkin et al., 2016; Richardson et al., 2020; Zhang et al., 2025). The aim is not to catalogue every reported CME-virus association, but to improve mechanistic interpretation, evidence grading and translational prioritization.

## 2 Literature search, study selection and evidence coding

This article was designed as a structured narrative review rather than a systematic review or meta-analysis. Searches were conducted in PubMed and Web of Science Core Collection from database inception to 18 June 2026; database-specific search strings, screening logic and record-management status are presented in **Supplementary Table S1**. The strategy combined terms related to CME machinery, including clathrin-coated pits, AP-2, epsin, CALM/AP180, dynamin, AAK1, GAK, auxilin and Hsc70, with terms related to viral entry, endocytosis, fusion, uncoating, genome release, inhibitors, natural products and host-directed antiviral modulation. Additional virus-specific and compound-specific searches, together with backward and forward citation tracking, were used to identify key mechanistic papers, older foundational studies and tool-specific reports relevant to **Tables 1** and **2**. Important omissions should not be interpreted as evidence against CME involvement. For example, Ebola virus was not included in **Table 1** because its principal initial uptake is generally assigned to macropinocytic pathways, although downstream TPC2- and NPC1-dependent endolysosomal checkpoints are highly relevant and are discussed separately in Section 7.

**Table 1:** Human-relevant and model viruses with reported CME or CME-associated entry in defined experimental models.

Virus and taxonomic context	Receptor/attachment/internalization factor(s)	Key CME-related finding	Evidence domains (P/G/I/F)	Particle/cell models and key caveat	Mechanistic classification; confidence	Entry stage resolved	References
Human immunodeficiency virus type 1 (HIV-1) *Retroviridae*; enveloped; +ssRNA-RT	CD4 with CCR5 or CXCR4; receptor/coreceptor usage determines tropism.	CME-associated uptake has been reported in permissive systems, but productive entry frequently occurs by plasma-membrane fusion and can involve alternative uptake routes.	P△/G△/I✓/F✓	HeLa-derived CD4+ cells, HeLaP4, 293T, TZM-bl and Rev-CEM. Limit conclusions to the tested strain, receptor/coreceptor usage and activation state.	CME-associated uptake; moderate, context-dependent confidence.	Attachment/CCP/CCV progression/internalization	(Daecke et al., 2005; Miyauchi et al., 2009; Gobeil et al., 2013; Herold et al., 2014; Li et al., 2017)
Vesicular stomatitis virus (VSV; zoonotic/model virus) *Rhabdoviridae*; enveloped; −ssRNA	LDLR and LDLR-family members.	Virions can induce *de novo* CCP formation and undergo productive clathrin-associated internalization in defined epithelial models.	P✓/G✓/I✓/F✓	HeLa, MDCK, BS-C-1 and related epithelial models. AP-2 and actin requirements can vary with model and particle geometry.	CME-dependent entry in defined epithelial models; high, model-defined confidence.	Internalization/endosomal delivery/productive infection	(Matlin et al., 1982; Le Blanc et al., 2005; Cureton et al., 2009, 2010; Johannsdottir et al., 2009; Finkelshtein et al., 2013)
Semliki Forest virus (SFV; historical/model alphavirus) *Togaviridae*; enveloped; +ssRNA	VLDLR and ApoER2 are established entry receptors in defined SFV models.	Classical studies support productive clathrin-associated uptake followed by low-pH-triggered early-endosomal entry.	P△/G✓/I✓/F✓	BHK-21 and related permissive models. Historical/model evidence should not be generalized to all human target cells.	CME-dependent entry in defined alphavirus models; high, model-defined confidence.	Endosomal delivery/fusion/penetration	(Helenius et al., 1980; Marsh et al., 1983; Clark et al., 2022; Cao et al., 2023; Yang et al., 2024)
Sindbis virus (SINV) *Togaviridae*; enveloped; +ssRNA	GAGs and cell-type-specific attachment/entry factors.	Low-pH-triggered endosomal entry and fusion are supported, but direct stage-resolved CME-specific evidence remains limited.	P△/G–/I△/F✓	HeLa, BHK-21 and related permissive cells. Treat as endosomal entry unless stronger CME-specific stage-resolved evidence is available.	CME-associated/low-pH endosomal entry; low, hypothesis-generating confidence.	Internalization/endosomal delivery	(Abell and Brown, 1993; DeTulleo and Kirchhausen, 1998; Glomb-Reinmund and Kielian, 1998; Smit et al., 1999)
Dengue virus (DENV) *Flaviviridae*; enveloped; +ssRNA	GAGs, DC-SIGN and other cell-type-specific attachment/entry factors.	CME supports entry in several serotype-cell systems, but clathrin-independent uptake is also documented.	P✓/G✓/I✓/F✓	Vero, HepG2 and other mammalian cells. Route usage varies by serotype and cell context.	CME-involved entry in selected systems; moderate, context-dependent confidence.	Internalization/productive infection	(van der Schaar et al., 2008; Acosta et al., 2009; Peng et al., 2009; Suksanpaisan et al., 2009; Cruz-Oliveira et al., 2015; Piccini et al., 2015; Ho et al., 2017)
Hepatitis C virus (HCV) *Flaviviridae*; enveloped; +ssRNA	HSPGs/GAGs, LDLR, SR-BI, CD81, CLDN1, OCLN and additional entry cofactors.	Clathrin-associated internalization and early-endosomal delivery are supported in HCVcc/HCVpp hepatoma systems.	P✓/G✓/I△/F✓	Huh7/Huh7.5 and derivative hepatoma cells. Specify HCVcc, HCVpp or other viral systems.	CME-involved entry in hepatoma systems; moderate, context-dependent confidence.	Internalization/endosomal uncoating	(Blanchard et al., 2006; Meertens et al., 2006; Helle and Dubuisson, 2008; Inoue et al., 2018)
Human rhinovirus 2 (HRV2; minor group) *Picornaviridae*; non-enveloped; +ssRNA	LDLR-family receptors.	A well-characterized minor-group rhinovirus model links LDLR-mediated uptake to clathrin-associated internalization and endosomal uncoating.	P✓/G✓/I✓/F✓	HeLa and related susceptible cells. Interpretation is specific to minor-group HRV2/LDLR.	CME-dependent LDLR-mediated uptake; high, model-defined confidence.	Internalization/capsid uncoating/productive infection	(Bayer et al., 2001; Snyers et al., 2003; Brabec-Zaruba et al., 2009)
Mammalian orthoreovirus (MRV; human-associated isolates) *Spinareoviridae*; non-enveloped; segmented dsRNA	Sialylated glycans/JAM-A support attachment; β1 integrin primarily supports post-attachment internalization and clathrin recruitment.	β1-integrin engagement can recruit clathrin and support post-attachment internalization after JAM-A/glycan-mediated attachment.	P△/G✓/I✓/F✓	BS-C-1, L929, GCF and related models. Specify strain/isolate origin and avoid broad extrapolation.	CME-involved internalization in defined MRV systems; moderate-to-high, model-defined confidence.	CCP formation/internalization/fusion	(Maginnis et al., 2008; Schulz et al., 2012; Koehler et al., 2021)
Influenza A virus (IAV) *Orthomyxoviridae*; enveloped; segmented −ssRNA	Sialic-acid-containing glycans; receptor/adaptor context can include epsin-1.	Individual virions can induce virus-centered CCPs, but productive entry can also proceed through alternative pathways.	P✓/G✓/I✓/F✓	HeLa, MDCK, BS-C-1 and other epithelial cells. Route usage depends on strain, cell type and multiplicity.	CME-involved entry in defined epithelial models; high, model-defined confidence.	Internalization	(Matlin et al., 1981; Sieczkarski and Whittaker, 2002; Rust et al., 2004; Sun et al., 2017; Du et al., 2024)
JC polyomavirus (JCPyV) *Polyomaviridae*; non-enveloped; circular dsDNA	LSTc-containing sialylated glycans; 5-HT2R and β-arrestin-linked entry cofactors.	Clathrin-associated uptake is supported in JCPyV-permissive systems, with β-arrestin contributing to internalization.	P✓/G✓/I✓/F✓	HeLa-H1, SVG-A and glial-lineage models. Distinguish JCPyV from other polyomaviruses.	CME-involved uptake in defined permissive systems; moderate-to-high, model-defined confidence.	Internalization/productive infection	(Pho et al., 2000; Elphick et al., 2004; Querbes et al., 2004, 2006; Neu et al., 2010; Assetta et al., 2013; Mayberry et al., 2019)
Hantaan virus (HTNV) *Hantaviridae*; enveloped; segmented −ssRNA	Reported factors include αvβ3 integrin, TIM-family phosphatidylserine receptors and other candidate host factors.	Clathrin-associated and dynamin-sensitive entry is reported, but receptor assignment and pathway requirements remain heterogeneous.	P△/G△/I△/F✓	Vero E6, A549, Jurkat T and related models. Explicitly distinguish authentic-virus from pseudotyped-particle studies.	CME-associated entry; moderate, particle-system- and cell-context-dependent confidence.	Productive infection	(Gavrilovskaya et al., 1999; Jin et al., 2002; Mou et al., 2006; Gu et al., 2024; Ma et al., 2024)
Crimean-Congo hemorrhagic fever virus (CCHFV) *Nairoviridae*; enveloped; segmented −ssRNA	LDLR and other host factors have been implicated.	Early infection is sensitive to endocytic/cholesterol perturbation, but direct stage-resolved clathrin/AP-2 evidence remains limited.	P✓/G△/I△/F✓	Vero E6 and other permissive cells. Treat receptor and CME assignments as model-defined.	CME-associated entry; low-to-moderate, context-dependent confidence.	Internalization/endosomal delivery	(Simon et al., 2009; Garrison et al., 2013; Shtanko et al., 2014; Xu et al., 2024)
Borna disease virus 1 (BoDV-1/BDV) *Bornaviridae*; enveloped; −ssRNA	Host receptor not definitively established; viral glycoprotein G mediates attachment.	Experimental evidence supports a clathrin-associated, caveola-independent endocytic pathway in tested permissive models.	P✓/G✓/I△/F✓	OL, Vero E6, Huh-7 and neuronal/glial models. Receptor identity remains unresolved.	CME-involved entry in tested models; moderate, context-dependent confidence.	Internalization/endosomal entry vs surface fusion	(Clemente and de la Torre, 2009)
SARS-CoV-2 *Coronaviridae*; enveloped; +ssRNA	ACE2; TMPRSS2 and cathepsins influence surface-fusion versus endosomal entry.	Clathrin-associated spike/virus uptake occurs in some models, but productive entry can shift to TMPRSS2-driven surface fusion or other endosomal routes.	P✓/G✓/I△/F✓	ACE2-expressing epithelial systems; pseudotype/authentic-virus and protease context must be specified.	CME-associated endosomal entry in selected systems; moderate, protease- and cell-context-dependent confidence.	Internalization/productive infection	(Hoffmann et al., 2020; Bayati et al., 2021; Koch et al., 2021)
Human coronavirus 229E (HCoV-229E) *Coronaviridae*; enveloped; +ssRNA	Aminopeptidase N (APN/CD13) is the canonical receptor.	CME perturbation and AP-2 μ-subunit knockout support CME involvement as one entry route in Huh-7 cells.	P✓/G✓/I△/F✓	Evidence is mainly from Huh-7 cells; respiratory epithelial models and protease-dependent route selection require separate interpretation.	CME-involved entry in Huh-7 cells; moderate, model-limited confidence.	Internalization	(Kawase et al., 2009; Bertram et al., 2013; Shirato et al., 2018; Andreu et al., 2024)
Zika virus (ZIKV) *Flaviviridae*; enveloped; +ssRNA	AXL, TIM/TAM factors, HSPGs and other cell-specific attachment/entry cofactors.	In T98G cells, pharmacological and CHC-siRNA perturbation supports CME involvement, but alternative uptake routes remain possible.	P✓/G✓/I△/F✓	Human neural-cell evidence is mainly derived from T98G cells; caveola-related perturbations also affect entry.	CME-involved entry in a T98G model; moderate, context-dependent confidence.	CCP/CCV progression/internalization/infectious entry	(Hamel et al., 2015; Hastings et al., 2017; Meertens et al., 2017; Persaud et al., 2018; Owczarek et al., 2019; Li et al., 2020)
West Nile virus (WNV) *Flaviviridae*; enveloped; +ssRNA	HSPGs, DC-SIGN/DC-SIGNR, αvβ3 integrin and other reported cell-dependent factors.	Dominant-negative Eps15, clathrin-pathway perturbation and immunoelectron microscopy support clathrin-associated infectious entry.	P✓/G✓/I✓/F✓	Permissive mammalian models; primary human target-cell validation remains limited.	CME-involved entry with stage-supported evidence; high, model-defined confidence.	Internalization/endosomal delivery	(Chu and Ng, 2004a, 2004b; Davis et al., 2006)
Japanese encephalitis virus (JEV) *Flaviviridae*; enveloped; +ssRNA	HSPGs, laminin receptor, integrins and other cell-context-dependent cofactors.	Clathrin/dynamin/Rab-dependent entry is reported in Vero/BHK-21 models, whereas clathrin-independent mechanisms occur in other cells.	P✓/G✓/I△/F✓	Vero/BHK-21 versus neuronal/fibroblast models; conclusions should remain cell-type specific.	CME-involved entry in Vero/BHK-21 models; moderate, cell-context-dependent confidence.	Internalization/fusion/penetration	(Nawa et al., 2003; Kalia et al., 2013; Liu et al., 2017; Yun and Lee, 2018)
Chikungunya virus (CHIKV) *Togaviridae*; enveloped; +ssRNA	MXRA8, GAGs and other attachment/entry cofactors.	Live single-virus tracking links many fusion-competent particles to clathrin-associated uptake before fusion, but alternative routes occur in some cells.	P✓/G△/I✓/F✓	Mammalian live-cell imaging models; strain, receptor usage and host-cell context matter.	CME-involved entry in imaging-defined models; moderate, context-dependent confidence.	Attachment/internalization/productive infection	(Bernard et al., 2010; Hoornweg et al., 2016; Zhang et al., 2018; Lee et al., 2019; Feng et al., 2023)
Human adenovirus type 2/5 (HAdV-2/5) *Adenoviridae*; non-enveloped; dsDNA	CAR mediates attachment; αv integrins promote internalization.	Dominant-negative clathrin/Eps15 and dynamin perturbation link integrin engagement to clathrin-associated internalization.	P△/G✓/I✓/F✓	Human epithelial, HeLa and HEK models. Distinguish CAR-mediated attachment from integrin-driven internalization.	CME-involved internalization after integrin engagement; moderate, context-dependent confidence.	Internalization/infectious entry	(Wickham et al., 1993; Bergelson et al., 1997; Wang et al., 1998; Meier et al., 2002)
Enterovirus A71 (EV-A71) *Picornaviridae*; non-enveloped; +ssRNA	SCARB2, PSGL-1 and other cell-type-dependent attachment/entry factors.	Genetic, imaging and inhibitor studies identify clathrin/AP-2 machinery as important for infectious entry in susceptible human-cell models.	P✓/G✓/I✓/F✓	Human rhabdomyosarcoma and other susceptible cells. Alternative ARF6/endophilin/caveolar routes occur in specific models.	CME-involved entry in susceptible human-cell models; moderate-to-high, context-dependent confidence.	Internalization	(Hussain et al., 2011; Lin et al., 2013; Chen et al., 2019; Zhu et al., 2023)
Rabies virus (RABV) *Rhabdoviridae*; enveloped; −ssRNA	nAChR, NCAM, p75NTR, TfR1, NRP2 and other reported neuronal or epithelial entry factors.	Clathrin-associated uptake is supported by imaging and genetic/kinase evidence involving AAK1/AP2M1 and receptor-coupled endocytosis.	P✓/G✓/I✓/F✓	BS-C-1, HEK293, N2a and primary neuronal models. Human neuronal validation remains comparatively limited.	CME-involved entry in epithelial/neuronal models; moderate, context-dependent confidence.	Internalization/productive infection vs surface fusion	(Lafon, 2005; Piccinotti et al., 2013; Wang et al., 2019, 2023; Sun et al., 2025)

P, pharmacological evidence; G, genetic evidence; I, imaging evidence; and F, stage-resolved functional evidence. ✓ indicates direct or sufficiently convergent support, △ indicates partial, indirect, or context-limited support, and – indicates that the criterion was not demonstrated or was not adequately assessed. These symbols are descriptive rather than numerical and should not be interpreted as an additive score; fulfillment of multiple evidence domains does not by itself establish CME-dependent entry. Detailed evidence supporting each designation, including supporting studies, experimental models, entry stage, alternative routes and the independent author-coding fields, is provided in **Supplementary Table S2**. Mechanistic classification and evidence confidence are reported as distinct dimensions within the same table field. Model-, strain-, receptor/cofactor-, particle-system-, cell- and assay-specific qualifiers should be considered when interpreting each entry. **Table 1** is intended as a representative mechanistic evidence map rather than a systematic prevalence inventory of CME use among viruses.

AAK1, adaptor-associated kinase 1; ACE2, angiotensin-converting enzyme 2; AP2A1, adaptor-related protein complex 2 subunit alpha 1; AP2M1, adaptor-related protein complex 2 subunit mu 1; APN, aminopeptidase N; ARF6, ADP-ribosylation factor 6; AXL, AXL receptor tyrosine kinase; CAR, coxsackievirus and adenovirus receptor; CCR5, C-C chemokine receptor 5; CD, cluster of differentiation; CLDN1, claudin-1; CXCR4, C-X-C chemokine receptor 4; DC-SIGN, dendritic cell-specific ICAM-3-grabbing non-integrin; DC-SIGNR, dendritic cell-specific ICAM-3-grabbing non-integrin-related protein; EEA1, early endosome antigen 1; Eps15, epidermal growth factor receptor pathway substrate 15; GAGs, glycosaminoglycans; GBP1, guanylate-binding protein 1; HCVcc, cell-culture-derived hepatitis C virus; HCVpp, hepatitis C virus pseudoparticles; HSPGs, heparan sulfate proteoglycans; LDLR, low-density lipoprotein receptor; LSTc, lactoseries tetrasaccharide c; MRV, mammalian orthoreovirus; MXRA8, matrix remodeling-associated protein 8; nAChR, nicotinic acetylcholine receptor; NCAM, neural cell adhesion molecule; NRP2, neuropilin 2; OCLN, occludin; p75NTR, p75 neurotrophin receptor; PSGL-1, P-selectin glycoprotein ligand-1; Rab, Ras-related GTPase; SCARB2, scavenger receptor class B member 2; SR-BI, scavenger receptor class B type I; TAM, Tyro3-AXL-Mer receptor tyrosine kinase family; TEM, transmission electron microscopy; TfR1, transferrin receptor 1; TIM-1, T-cell immunoglobulin and mucin domain 1; TMPRSS2, transmembrane serine protease 2; VLDLR, very-low-density lipoprotein receptor; 5-HT2R, 5-hydroxytryptamine 2 receptor.

**Table 2:** Host-directed strategies targeting CME-associated viral-entry vulnerabilities.

Host target/pathway	Representative intervention	Relevant virus/model	Entry-stage mechanism	Current evidence	Disease-relevant/*in vivo* evidence	Major translational limitation
ASM–sphingomyelin/ceramide axis	Fluoxetine and other FIASMAs	SARS-CoV-2, IAV, HAdV models	Membrane/endolysosomal organization; virus internalization and early-endosome trafficking in HAdV	Context-dependent pharmacological evidence; sphingomyelin dependence is not synonymous with ASM dependence	HAdV: age-linked human datasets and combination evidence with brincidofovir; other viruses mainly cell based	Virus-specific effects; pleiotropic lysosomal pharmacology; exposure and selectivity
TPC2-dependent endolysosomal trafficking	Tetrandrine/TPC-directed modulation	Ebola virus/filovirus models	Late-endosomal ion-dependent trafficking and fusion competence	Genetic knockout/siRNA plus pharmacological inhibition support TPC dependence	Human macrophage inhibition and therapeutic efficacy in mouse EBOV model	Host physiology, target selectivity, systemic exposure and breadth across virus families
NPC1/cholesterol homeostasis	NPC1-directed or cholesterol-redistributing approaches	Ebola virus and related filovirus models	Late-endosomal intracellular receptor function and fusion/escape competence	Strong genetic and mechanistic evidence for NPC1; cholesterol-redistributing drugs provide supportive pharmacology	Preclinical; NPC1 is essential *in vivo* for ebolavirus pathogenesis	Essential cholesterol homeostasis; distinguish direct NPC1 effects from broader lipid redistribution
OSBP/ORP-dependent lipid transport	Itraconazole	Enteroviruses	OSBP/ORP4-dependent lipid transfer at replication organelles; not an entry-stage CME mechanism	Strong target evidence for enterovirus RNA replication	Primarily cell-based antiviral evidence	Post-entry mechanism; should not be misclassified as NPC1/CME entry inhibition
PIKfyve-dependent endosomal maturation	Apilimod and related PIKfyve inhibitors	Filoviruses and SARS-CoV-2-related models	Endosomal phosphoinositide remodeling, maturation and fusion competence	Pharmacological and genetic support in selected systems	Limited translational validation compared with mechanistic evidence	Essential endolysosomal functions, exposure and toxicity
AAK1/GAK/NAK-regulated trafficking	Kinase-directed modulation/selected repurposing strategies	RABV, SARS-CoV-2 and other virus models	Adaptor phosphorylation and intracellular/endocytic trafficking	Context-dependent genetic/pharmacological evidence	Variable; much evidence remains model specific	Broad kinase pharmacology and off-target effects; direct CME blockade should not be inferred

The strategies summarized here represent host-directed approaches that perturb virus-relevant entry or endosomal checkpoints rather than selective inhibitors of CME as a whole. Evidence strength and translational maturity differ substantially among targets. Entries supported primarily by cell-culture studies should not be interpreted as validated antiviral therapies. Disease-relevant or *in vivo* evidence is indicated separately where available.

GAK, cyclin G-associated kinase; NAK, numb-associated kinase; PIKfyve, phosphoinositide kinase, FYVE-type zinc finger containing; V-ATPase, vacuolar-type H+-ATPase.

Records were screened for mechanistic relevance rather than counted toward a quantitative prevalence estimate of CME usage among viruses. Original experimental studies were prioritized when they directly examined viral attachment, internalization, clathrin-associated uptake, endosomal delivery, fusion, uncoating, genome release or early productive infection in relation to CME or CME-associated pathways. Mechanistic studies of CME machinery, pharmacological perturbants, genetic perturbation approaches and host-directed entry modulators were included when they informed interpretation of viral-entry experiments. Selected authoritative reviews were cited only to support general concepts, methodological limitations or translational framing.

Representative viruses were selected for **Table 1** when the literature provided mechanistically informative evidence linking a CME-specific host factor or clathrin-positive structure to an entry-stage event and when the virus fulfilled at least one of the following criteria: (i) a historically influential model of endocytic viral entry; (ii) substantial relevance to human disease; (iii) availability of stage-resolved mechanistic evidence; (iv) a receptor- or adaptor-specific mechanism informative for CME biology; or (v) well-documented context-dependent switching between CME and alternative entry routes. Viruses supported predominantly by nonspecific inhibitor sensitivity, dynamin dependence, low-pH sensitivity or endpoint infection measurements were not prioritized for the main table. **Table 1** should therefore be interpreted as a purposefully selected mechanistic map rather than an exhaustive inventory of viruses reported to use CME.For transparency, **Table 1** summarizes four complementary evidence domains—pharmacological (P), genetic (G), imaging (I), and stage-resolved functional (F) evidence—whereas the study-level basis for each designation is provided in **Supplementary Table S2**.

Two reviewers (QW and YX) independently assessed all virus/model entries contributing to **Table 1** using the prespecified evidence framework. The assessments were completed independently before comparison. Disagreements were resolved by consensus, with JY serving as adjudicator when required. The extracted domains included particle system, receptor/cofactor context, host-cell model, pharmacological, genetic, imaging and stage-resolved functional evidence, the entry stage resolved, major alternative routes, mechanistic classification and evidence confidence. Detailed coding and final adjudicated classifications are provided in **Supplementary Table S2**.

Evidence interpretation was stage-resolved and context-specific. Evidence based only on dynamin dependence, low-pH sensitivity, endosomal acidification inhibitors, broad trafficking perturbation, static colocalization or endpoint infection reduction was not considered sufficient to assign strict CME-dependent entry. Strict CME-dependent entry was reserved for defined virus-receptor-cell contexts in which direct perturbation of core or proximal CME machinery, supported by rescue or an orthogonal approach, was linked to stage-resolved evidence of productive virion progression through clathrin/AP-2-positive CCPs or CCVs and to an early functional readout of internalization, penetration, fusion, capsid uncoating or genome release. Pharmacological findings were considered supportive but were not required when stronger direct and orthogonal mechanistic evidence was available. Evidence was downgraded when viability, receptor expression, viral binding, representative CME cargo uptake, alternative endocytic routes or post-entry effects were not adequately controlled.

No formal risk-of-bias assessment, PRISMA flow diagram or quantitative meta-analysis was undertaken because this review was designed as a structured narrative review. Database searches were used to identify and triangulate mechanistically relevant evidence rather than to estimate the prevalence of CME usage among viruses. Database record counts were not used as inclusion metrics and were not retrospectively reconstructed as systematic-review denominators. The synthesis should therefore be interpreted as an evidence-graded mechanistic review rather than as a numerical inventory of CME-dependent viruses.

## 3 Biological determinants and stage-resolved mechanisms of CME-associated viral entry

CME is a conserved route by which selected plasma-membrane cargoes are internalized through clathrin-coated pits (CCPs) and delivered to endosomal compartments (Kaksonen and Roux, 2018; McMahon and Boucrot, 2011; Mettlen et al., 2018). For viral-entry studies, CME is best analyzed as a series of experimentally separable checkpoints—CCP nucleation, cargo selection, coat assembly and membrane curvature, dynamin-dependent scission, coat uncoating and endosomal delivery—as summarized in **Figure 1**. This distinction is critical because reduced endpoint infection may reflect impaired attachment, internalization, endosomal maturation, fusion, capsid remodeling, genome release or later replication-associated events (Yamauchi and Helenius, 2013; Rennick et al., 2021). Evidence for CME-dependent entry should therefore identify the affected entry stage and demonstrate that virions physically and functionally progress through clathrin-positive structures in a defined virus-receptor-cell context.

**Figure 1:**
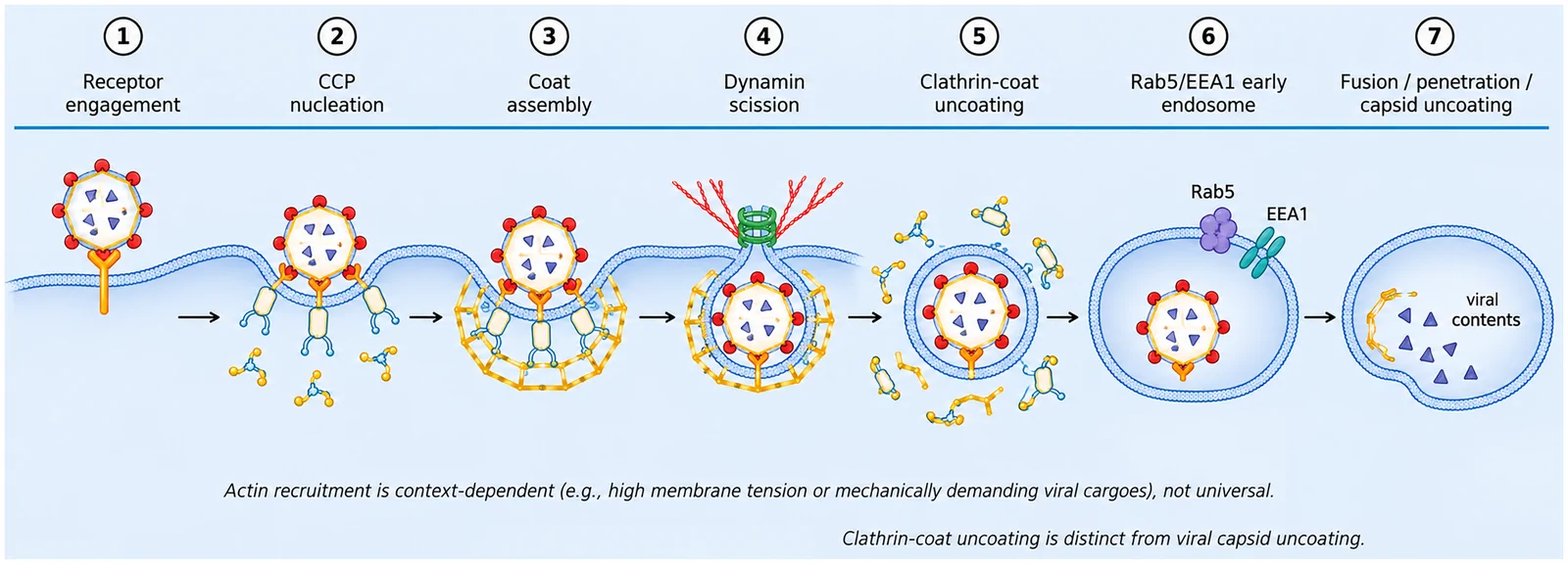
Stage-resolved and context-dependent checkpoints of CME-associated viral entry. Virus–receptor complexes may engage CME through receptor/adaptor-dependent cargo recruitment, CCP nucleation and coat assembly, followed by dynamin-dependent scission and clathrin-coat disassembly. Uncoated endocytic carriers subsequently deliver viral cargo to Rab5/EEA1-positive early endosomes, from which virus-specific fusion, penetration, capsid-uncoating or genome-release events may proceed. Actin recruitment is context dependent and may become important for mechanically demanding viral cargoes or elevated membrane tension; it is not an obligatory component of every CME event. Clathrin-coat uncoating is distinct from viral capsid uncoating.

## 4 Receptor organization and cargo-specific adaptor recruitment

Viral engagement of CME is determined not only by the presence of an entry receptor but also by how receptor-bound virions reorganize the plasma membrane and recruit cargo-specific endocytic machinery. Receptor clustering can locally increase the probability of CCP initiation, whereas the cytoplasmic domains of receptors or receptor-associated proteins can recruit AP-2 and alternative adaptors that connect extracellular virions to the clathrin coat. This relationship is highly cargo specific. Influenza A virus can recruit epsin 1 as a cargo-specific adaptor and induce *de novo* CCP formation beneath individual virions, whereas JC polyomavirus uses beta-arrestin-dependent signaling to promote clathrin-mediated internalization. Mammalian orthoreovirus provides another example in which post-attachment engagement of beta1 integrin promotes clathrin recruitment, and rabies virus entry has been linked to AAK1/AP2M1-regulated endocytic signaling. These examples indicate that viral use of CME is actively shaped by receptor organization and adaptor recruitment rather than representing passive capture by constitutively forming coated pits (Chen and Zhuang, 2008; Liu et al., 2010; Mayberry et al., 2019; Wang et al., 2019; Koehler et al., 2021).

### 4.1 Nucleation and coat initiation

CME begins at PI(4,5)P2-enriched plasma-membrane regions where early endocytic proteins assemble CCP initiation platforms. FCHO1/2, AP-2, Eps15/Eps15R and intersectins cooperate through multivalent interactions to recruit clathrin and additional coat-associated factors (Traub, 2009; Henne et al., 2010; Liu et al., 2010; McMahon and Boucrot, 2011; Nunez et al., 2011; Taylor et al., 2011; Umasankar et al., 2012; Zaccai et al., 2022; Jin et al., 2024). For viruses, nucleation-stage evidence asks whether receptor-bound virions trigger *de novo* CCP formation or are captured by pre-existing CCPs. Proximity to clathrin is not enough; stronger evidence requires live-cell imaging of CCP initiation beneath bound virions, recruitment of early CME markers to virus-binding sites and acute perturbation showing reduced early internalization without simply reducing attachment (Rennick et al., 2021).

### 4.2 Cargo selection

Cargo selection links receptor engagement to coat recruitment. AP-2 is the best-characterized CME adaptor, whereas epsin, CALM/AP180, Eps15 and other accessory proteins can contribute to receptor recruitment, CCP stabilization and cargo-specific internalization (Benmerah et al., 1999; Ford et al., 2001; Wendland, 2002; Mukhopadhyay and Riezman, 2007; Schmid and McMahon, 2007; Traub and Bonifacino, 2013). Viruses usually do not provide cytoplasmic sorting motifs themselves; instead, they enter CME indirectly by binding host receptors, co-receptors or attachment factors whose cytoplasmic tails or associated proteins connect the virus-receptor complex to endocytic machinery (Maginnis, 2018). Evidence for cargo-selection involvement should combine receptor-specific perturbation, adaptor knockdown or rescue, quantitative recruitment of virions to AP-2/clathrin-positive CCPs and controls showing that reduced infection is not merely caused by impaired receptor expression or viral binding (Grove and Marsh, 2011; Yamauchi and Helenius, 2013).

### 4.3 Coat assembly and membrane curvature

After nucleation and cargo recruitment, clathrin and adaptor proteins assemble into a maturing coat around virus-receptor-containing membrane domains. Epsin and other curvature-associated proteins may help deform or stabilize the membrane, whereas Eps15 mainly functions as an endocytic accessory or scaffold protein rather than as a primary curvature-generating factor (Tebar et al., 1996; Ford et al., 2002; Wakeham et al., 2003; Fotin et al., 2004b; McMahon and Boucrot, 2011; Kaksonen and Roux, 2018). Models of CCP curvature formation differ in whether curvature develops during assembly or after an initially flatter lattice is remodeled. For viral entry, the practical question is whether the virion-containing pit matures toward vesicle formation rather than remaining an abortive or nonproductive clathrin-positive structure (Mettlen et al., 2018).

Because virions are larger and mechanically more complex than many physiological CME cargoes, they may stabilize CCPs, delay maturation or remain associated with abortive pits (Mettlen et al., 2018). Imaging evidence should therefore distinguish transient clathrin colocalization from productive CCP maturation. Useful readouts include virion-clathrin co-tracking, CCP lifetime, maturation probability, scission and early internalization. Coat-assembly involvement is best supported when perturbing coat maturation reduces early virion internalization rather than only endpoint infection.

### 4.4 Membrane lipids, particle geometry and context-dependent actin recruitment

Membrane composition and mechanics provide an additional layer of control over virus-associated CME. PI(4,5)P2 is required for the initiation and stabilization of CCPs, whereas cholesterol and sphingolipids influence membrane order, receptor organization, curvature and the biophysical environment encountered by enveloped viruses (Antonescu et al., 2011). These effects should not be interpreted as evidence for a single universal lipid requirement, because lipid dependence can occur at receptor binding, vesicle formation, endosomal trafficking or membrane fusion. Mechanical constraints are also particularly relevant to viral cargoes. Virions are substantially larger and more geometrically diverse than many physiological CME cargoes, and particle size or shape can alter coat completion and the requirement for accessory force-generating mechanisms. For example, the elongated morphology of vesicular stomatitis virus can produce incompletely coated carriers whose internalization requires actin assembly (Cureton et al., 2009, 2010). More generally, actin recruitment becomes particularly important when membrane tension or cargo geometry creates an energetic barrier to invagination and scission; it should therefore be regarded as a context-dependent accessory mechanism rather than an obligatory component of every mammalian CME event (Cureton et al., 2009; Boulant et al., 2011).

### 4.5 Dynamin-dependent scission

Mature CCPs are released from the plasma membrane by dynamin-dependent scission. Dynamin assembles at the neck of deeply invaginated pits and uses GTP hydrolysis to drive membrane remodeling and vesicle release (Danino et al., 2004; Bashkirov et al., 2008; Pucadyil and Schmid, 2008; Chappie et al., 2011; Boucrot et al., 2012; Ferguson and De Camilli, 2012; Mattila et al., 2015; Antonny et al., 2016; Kaksonen and Roux, 2018; Prichard et al., 2021). For viral entry, scission marks the transition from surface-associated virus-receptor complexes to intracellular vesicular cargo. Dynamin inhibitors, dominant-negative mutants and knockdown approaches are therefore useful for testing uptake requirements, but their interpretation must be stage resolved.

Dynamin dependence is not equivalent to CME dependence because several clathrin-independent pathways also require dynamin (Mayor et al., 2014). Dynamin-sensitive infection should be interpreted as evidence for dynamin-dependent uptake unless it is accompanied by concordant perturbation of clathrin, AP-2 or other CME components. Stronger scission-stage evidence should show that virions normally progress from clathrin/AP-2-positive CCPs into intracellular vesicular compartments and that this transition is disrupted when dynamin-dependent scission is acutely inhibited.

### 4.6 Coat uncoating and endosomal delivery

After scission, newly formed clathrin-coated vesicles must lose their coat before fusing with early endosomes. This coat-uncoating step is regulated mainly by Hsc70, auxilin or GAK, and PI(4,5)P2 dephosphorylation mediated by synaptojanin (Barouch et al., 1994; McPherson et al., 1996; Fotin et al., 2004a; Stricher et al., 2013; Sousa et al., 2016; Wang et al., 2020). In this review, “coat uncoating” refers to disassembly of the clathrin coat, not viral capsid uncoating or genome release.

For viruses, endosomal delivery connects CME-mediated internalization to receptor dissociation, acidification, proteolytic activation, membrane fusion, penetration, capsid remodeling and viral genome release. Sensitivity to bafilomycin A1, chloroquine, cathepsin inhibitors, PIKfyve inhibitors or broad trafficking modulators does not by itself prove CME dependence because these agents act downstream of CCP formation and can affect multiple endocytic routes (Grove and Marsh, 2011; Mayor et al., 2014). CME-associated endosomal delivery is better supported by combining early uptake assays, endosomal colocalization, fusion or capsid-uncoating reporters, time-of-addition experiments and quantification of surface-stripping-resistant internalized input genomes or, where replication is specifically assessed, newly synthesized viral RNA.

## 5 Virus-induced remodeling and entry-route plasticity

Viral entry can remodel the endocytic machinery rather than merely exploit a pre-existing pathway (Rust et al., 2004; Cureton et al., 2009; Koehler et al., 2021; Broich et al., 2025)Single-particle studies have shown that influenza virus, VSV and reovirus can stabilize or induce CCP formation at virus-binding sites, whereas recent nanoscale analyses of influenza virus have revealed broader local reorganization of the virus-cell interface. Such remodeling is superimposed on marked pathway plasticity. Changes in receptor abundance, protease availability, membrane composition, particle state, multiplicity of infection or host-cell differentiation can shift productive entry between CME, macropinocytosis, other clathrin-independent routes and plasma-membrane fusion. Consequently, pathway choice should be considered an emergent property of a defined virus-receptor-cell system rather than a fixed taxonomic property of a virus species (Ehrlich et al., 2004).

## 6 Evidence standards for assigning CME-dependent viral entry

Because CME stages are experimentally separable, evidence for CME-dependent viral entry should be assigned according to the entry step directly affected and the controls used to exclude non-entry effects. The following operational criteria are used throughout this review.

### 6.1 Pharmacological evidence

Pharmacological evidence can provide an initial indication of CME-associated viral entry, but it should not be treated as definitive on its own. Entry studies should assess cell viability, viral binding, early internalization, transferrin uptake and markers of alternative endocytic routes. Whenever possible, structurally distinct inhibitors should be used for cross-validation. For chlorpromazine, Pitstop 2, tyrphostin A23, dynasore, bafilomycin A1, chloroquine and PIKfyve inhibitors, potential cytotoxicity, off-target effects, cargo selectivity and post-entry effects should be explicitly considered (Yoshimori et al., 1991; Wang et al., 1993; Guinea and Carrasco, 1994; Banbury et al., 2003; Macia et al., 2006; von Kleist et al., 2011; Dutta et al., 2012; Gillespie et al., 2013; Willox et al., 2014; Daniel et al., 2015; Nelson et al., 2017; Mauthe et al., 2018; Kang et al., 2020; Riva et al., 2020; Altulea et al., 2021; Ou et al., 2021; Prichard et al., 2024).

### 6.2 Genetic evidence

Genetic evidence should preferentially involve short-term, validated perturbation of core CME components or virus-relevant receptors, including siRNA, CRISPR-based perturbation, dominant-negative mutants and rescue experiments targeting clathrin heavy chain (CHC), AP-2, Eps15, epsin, dynamin or relevant entry receptors (van der Bliek et al., 1993; Benmerah et al., 1999; Motley et al., 2003; Huang et al., 2004; Gilbert et al., 2014; Wang et al., 2014; Doench et al., 2016; Sanson et al., 2018). Long-term knockout or sustained silencing of essential endocytic machinery can trigger cellular adaptation or compensatory use of alternative pathways, so rescue, acute depletion and receptor-specific controls are particularly important.

### 6.3 Imaging evidence

Imaging evidence is essential for determining whether viral particles are spatially and temporally linked to CME structures during the entry window. Electron microscopy and related correlative or volume electron microscopy approaches can provide ultrastructural evidence that virions are associated with CCPs, CCVs or early endocytic compartments (Helenius et al., 1980; Chu and Ng, 2004a; Plitzko et al., 2009; Guerin and Lippens, 2021). These approaches validate membrane invagination, vesicle formation and high-resolution virion localization, but static snapshots cannot establish the complete sequence from attachment to internalization, endosomal delivery, fusion or viral uncoating.

Live-cell fluorescence imaging provides complementary temporal information. TIRFM, confocal microscopy, stimulated emission depletion microscopy and single-particle tracking of fluorescently labeled virions can monitor recruitment to clathrin/AP-2-positive CCPs, *de novo* CCP formation, maturation, scission and delivery to Rab5- or EEA1-positive endosomes (Rust et al., 2004; Brandenburg and Zhuang, 2007; van der Schaar et al., 2008; Ni et al., 2024; Broich et al., 2025). Interferometric scattering microscopy combined with fluorescence colocalization may further improve localization precision for early virus-CCP interactions (Kukura et al., 2009).

Nevertheless, imaging requires cautious interpretation. Static colocalization between virions and clathrin puncta may reflect transient proximity, abortive CCP trapping, post-binding accumulation or overlap with endosomal structures rather than productive clathrin-associated uptake. Stronger evidence should quantify virion recruitment frequency, CCP lifetime, maturation probability, internalization after scission and delivery to early endosomes, and should test whether acute perturbation of clathrin, AP-2, Eps15, epsin, dynamin or related factors disrupts the imaged entry step.

Several technical controls are also necessary. Virus labeling should preserve infectivity and receptor usage; tagged host proteins should not distort CME dynamics; and phototoxicity, overexpression artifacts, low signal-to-noise ratio and insufficient temporal resolution should be minimized. Imaging experiments should distinguish surface-bound from internalized virions by synchronized binding and warming, acid wash, protease stripping, antibody-accessibility assays or fluorescence-quenching strategies. Imaging evidence becomes most persuasive when paired with early internalization, fusion or penetration reporters, capsid-uncoating markers, quantification of surface-stripping-resistant internalized input genomes or, where replication is specifically assessed, newly synthesized viral RNA, and subsequent infectivity (Rust et al., 2004; Brandenburg and Zhuang, 2007; van der Schaar et al., 2008; Kukura et al., 2009; Ni et al., 2024; Broich et al., 2025).

### 6.4 Stage-resolved functional assays

Stage-resolved functional assays should distinguish attachment, internalization, fusion or penetration, viral capsid uncoating, genome release and post-entry replication. Common strategies include temperature-synchronized binding followed by warming-induced internalization, time-of-addition assays, acid wash or protease treatment to remove surface-bound virions, fusion or uncoating reporter systems, quantification of surface-stripping-resistant internalized input viral genomes and, where appropriate, assays specifically detecting newly synthesized viral RNA. CME involvement is strengthened when perturbation affects internalization or fusion/uncoating within the early entry window rather than merely reducing later replication or overall cell fitness (Mercer et al., 2010; Yamauchi and Helenius, 2013; Rennick et al., 2021).

### 6.5 Mechanistic classification and evidence confidence

Three operational terms are used to describe mechanistic status. ‘CME-associated entry’ denotes limited or hypothesis-generating evidence, such as inhibitor sensitivity, static clathrin colocalization or endpoint infection changes without resolution of the affected entry stage. ‘CME-involved entry’ requires concordant evidence from at least two independent approaches, including at least one CME-proximal perturbation or dynamic imaging approach, together with an entry-window functional effect and appropriate controls. This category indicates that CME contributes to productive entry but does not establish that it is obligatory or exclusive. ‘CME-dependent entry’ is reserved for contexts in which direct perturbation of core or proximal CME machinery, supported by rescue or orthogonal validation, is linked to productive stage-resolved virion progression through clathrin/AP-2-positive CCPs or CCVs and to an early functional entry readout. Mechanistic status is reported separately from evidence confidence, which is additionally influenced by model relevance, experimental controls, assay resolution and consistency across studies (**Figure 2**; **Table 3**).

**Figure 2:**
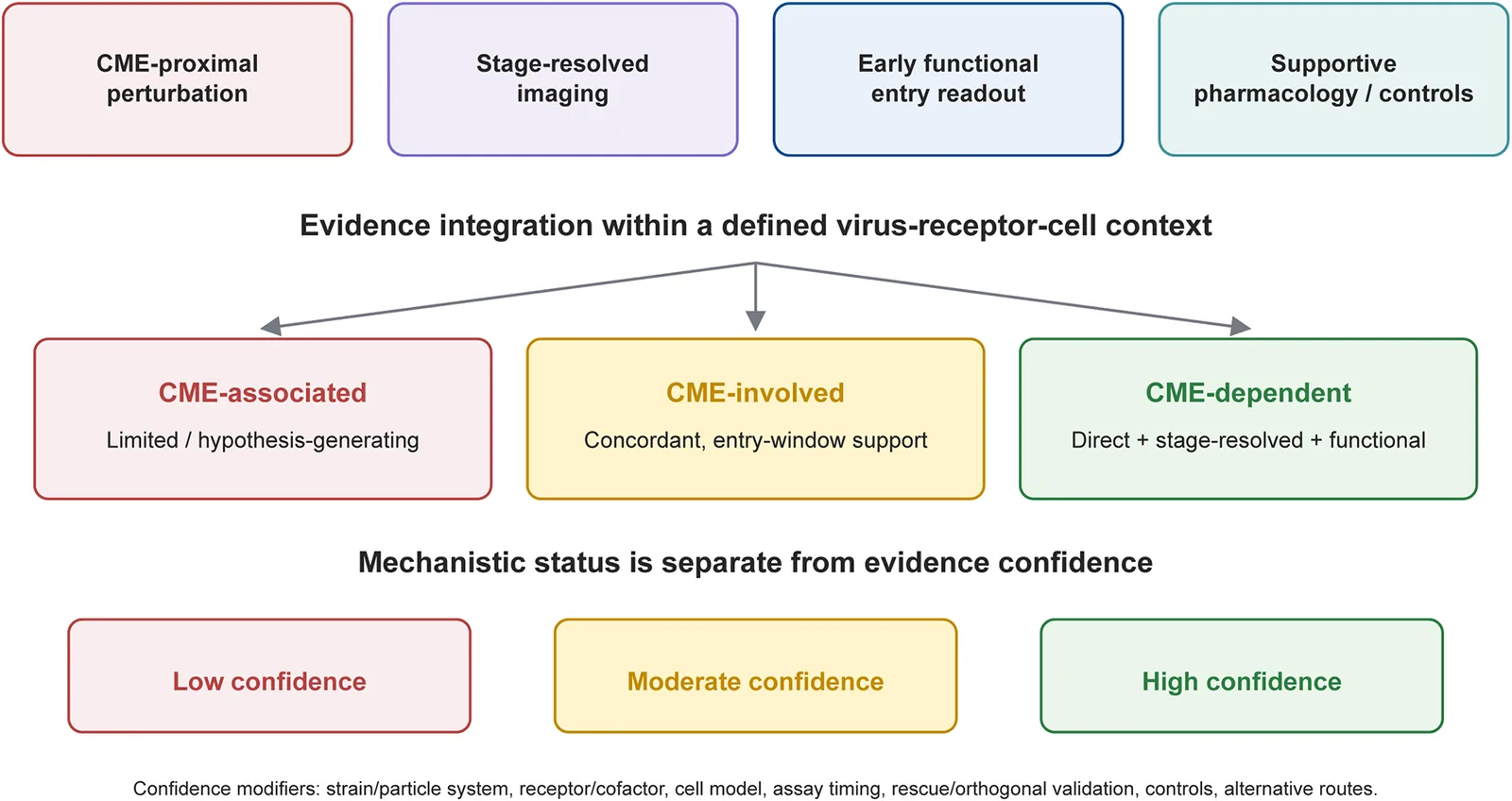
Context-dependent evidence framework for assigning CME-related viral entry. Mechanistic status (CME-associated, CME-involved or CME-dependent) is evaluated separately from evidence confidence. Strict CME-dependent entry requires direct perturbation of core or proximal CME machinery with rescue or orthogonal validation, stage-resolved evidence of productive virion progression through clathrin/AP-2-positive coated pits or vesicles, and an early functional entry readout in a defined virus–receptor–cell context. Pharmacological evidence is supportive rather than universally mandatory when stronger direct mechanistic evidence is available. Evidence confidence is additionally modified by viral strain or particle system, receptor/cofactor context, host-cell model, assay timing, essential controls and alternative entry routes.

**Table 3:** Operational evidence standards for assigning CME-associated, CME-involved and CME-dependent viral entry.

Evidence category	Minimum evidence	Required controls and validation	Recommended wording
CME-associated entry	Single or incomplete evidence, including inhibitor sensitivity, static clathrin colocalization, dynamin dependence or endpoint infection reduction without stage resolution.	Report viability, viral binding and representative cargo-uptake controls where available; do not infer the affected entry step from endpoint infection alone.	“CME-associated entry/uptake was suggested”.
CME-involved entry	Concordant evidence from at least two independent approaches, including at least one CME-proximal perturbation or dynamic imaging approach, linked to an entry-window functional effect.	Control viability, viral binding, receptor expression and major alternative uptake routes; use orthogonal validation or rescue when feasible.	“CME contributes to entry in this defined model”.
CME-dependent entry	Direct perturbation of core/proximal CME machinery with rescue or orthogonal validation, plus stage-resolved productive virion progression through clathrin/AP-2-positive CCPs/CCVs and an early functional entry readout.	Specify strain/particle system, receptor/cofactor, cell model and assay window; exclude major alternative explanations and post-entry effects. Pharmacology is supportive rather than universally mandatory.	“CME-dependent entry in this defined virus-receptor-cell context”.

## 7 Viruses with reported CME or CME-associated entry pathways

Semliki Forest virus (SFV) is one of the earliest and best-characterized enveloped animal-virus models of productive clathrin-associated endosomal entry (Helenius et al., 1980), and subsequent work has shown that diverse viruses can exploit CME or CME-associated endosomal entry. Classical VSV and SFV models illustrate rapid internalization and low-pH triggering on a minute time scale, but the exact timing depends on the viral particle, receptor, cell type and synchronization conditions (Mercer et al., 2010). The purpose of **Table 1** is therefore not to rank viruses by general CME dependence, but to map the strength and context of CME-related evidence.

Cargo can actively influence CCP assembly rather than simply entering constitutive pits. This is particularly clear for VSV, reovirus and influenza A virus, which can induce *de novo* CCP assembly at attachment sites (Ehrlich et al., 2004; Rust et al., 2004; Cureton et al., 2009; Johannsdottir et al., 2009; Koehler et al., 2021). At the same time, AP-2 dependence is not universal: some cargoes or viruses can undergo partial AP-2-independent internalization through alternative adaptors (Conner and Schmid, 2003; Robinson, 2004). These examples support the need for virus-receptor-cell-specific wording rather than generalized CME labels.

**Table 1** applies the evidence framework defined above to representative human-relevant and model viruses with reported CME or CME-associated entry. Evidence levels should be read together with the cell-model and caveat column because “Strong, model-defined” denotes strong evidence within specified experimental systems, not universal dependence across all strains, receptors or host cells.

## 8 Experimental tools and host-directed modulators used to interrogate CME-associated viral entry

Chemical and peptide perturbants are useful for dissecting viral-entry pathways, but they should be described according to the step they interrogate rather than as tools that uniformly block CME. Reduced viral entry or infection after compound treatment does not identify clathrin-coat initiation, adaptor recruitment or coated-pit maturation unless binding, early internalization, fusion/uncoating and cell-viability controls are included (Macia et al., 2006; von Kleist et al., 2011; Dutta et al., 2012; Willox et al., 2014; Rennick et al., 2021; Prichard et al., 2024; Zhang et al., 2025).

The tools reviewed here are therefore separated into functionally distinct categories. Direct or proximal CME perturbants affect clathrin/adaptor interactions, cargo recruitment, coated-pit dynamics or coat uncoating. Dynamin inhibitors test vesicle-scission dependence but cannot distinguish CME from clathrin-independent dynamin-dependent pathways. Host-kinase and trafficking modulators influence AAK1/GAK-regulated trafficking or broader kinase networks. Endosomal acidification, maturation and trafficking modulators interrogate downstream entry steps that may be shared by multiple uptake routes. Natural products and derivatives should be regarded as chemical probes or model-specific leads unless target engagement, selectivity, reversibility and *in vivo* efficacy are established.

Older global perturbations, including cytosolic acidification, potassium depletion and hypertonic treatment, were historically important for implicating coated-pit uptake (Carpentier et al., 1989; Cosson et al., 1989; Hansen et al., 1993). Likewise, clathrin or dynamin knockdown and dominant-negative mutants remain valuable mechanistic tools (van der Bliek et al., 1993; Deborde et al., 2008; Bhave et al., 2020; Chen et al., 2020). However, global physicochemical treatments lack pathway specificity, and long-term genetic perturbation may remodel endocytic pathways. These approaches should therefore support, but not replace, stage-resolved evidence.

Pharmacological tools should therefore be interpreted according to their experimentally demonstrated target and entry stage, with concentration-response, viability, cargo-uptake and orthogonal-validation controls reported wherever possible. Detailed assay-specific concentrations, value types, cell models and exposure conditions are provided in **Supplementary Table S3**; the main-text **Table 2** instead summarizes host-directed strategies with potential translational relevance.

## 9 Host-directed therapeutic opportunities and translational constraints

### 9.1 Membrane sphingolipids and the ASM-ceramide axis

Membrane sphingolipids represent a potentially tractable host vulnerability because they regulate receptor organization, membrane order and endolysosomal function without necessarily requiring sustained blockade of the core clathrin machinery. Acid sphingomyelinase (ASM) converts sphingomyelin to ceramide and can thereby alter membrane-domain organization and endolysosomal trafficking. Functional inhibitors of ASM (FIASMAs), including fluoxetine, have shown antiviral activity in cell-culture models of SARS-CoV-2 and influenza A virus, with fluoxetine-associated effects including impaired endolysosomal acidification and cholesterol redistribution (Schloer et al., 2020). However, sphingomyelin dependence should not be equated with universal ASM dependence. In influenza A virus, depletion of cellular or virion-associated sphingomyelin impaired attachment and entry, whereas pharmacological ASM inhibition with desipramine and genetic ASM deficiency did not reproduce this effect, indicating that membrane sphingomyelin itself and ASM activity can make distinct contributions (Audi et al., 2020). Recent evidence further identifies ASM as an age-dependent host determinant of adenovirus internalization and early-endosomal trafficking and suggests that pharmacological ASM modulation may complement direct-acting antiviral therapy (Post et al., 2026). Age-related changes in ASM expression and activity may therefore modify membrane and endolysosomal environments that influence viral susceptibility. However, the contribution of this axis to age-associated severity is likely to be virus specific and should not be generalized from adenovirus entry to severe viral disease without direct mechanistic evidence. These observations nevertheless raise the possibility that host age may influence both the efficacy and the therapeutic window of ASM-directed antiviral interventions. These findings support the ASM-ceramide-sphingomyelin axis as a context-dependent host-entry vulnerability rather than as a universal inhibitor of CME. Fluoxetine-associated inhibition of Ebola virus has also been linked to endolysosomal cholesterol redistribution; however, this finding is better interpreted as lipid/endolysosomal remodeling than as definitive evidence for an ASM-specific requirement during Ebola virus entry (Kummer et al., 2022).

### 9.2 TPC2-dependent endolysosomal trafficking

Two-pore channels, particularly TPC2, illustrate how a downstream endolysosomal checkpoint can be targeted without directly inhibiting CCP formation. TPC proteins regulate ion-dependent endosomal trafficking and have been implicated in the progression of internalized virions through late endosomal compartments. In Ebola virus models, genetic disruption or pharmacological inhibition of TPC function arrested endosomal virus trafficking and inhibited infection, and tetrandrine showed antiviral activity in human macrophages and experimental mouse models (Sakurai et al., 2015). Importantly, this mechanism should be classified as an endolysosomal entry vulnerability rather than a direct CME target, because Ebola virus uptake can occur through macropinocytic pathways before TPC-dependent trafficking. TPC2 therefore exemplifies the broader principle that therapeutically accessible host dependencies may reside downstream of initial internalization and may be shared by more than one uptake route. Selectivity, systemic physiological functions and the extent of antiviral activity across virus families remain important translational limitations.

### 9.3 Cholesterol trafficking, NPC1 and related lipid-transport vulnerabilities

Endosomal cholesterol distribution is another determinant of membrane trafficking and fusion competence. Niemann-Pick C1 (NPC1) is particularly important for filovirus entry because cleaved Ebola virus glycoprotein engages NPC1 in late endosomal compartments, making NPC1 both an intracellular receptor and a mechanistically defined host dependency (Carette et al., 2011). Pharmacological perturbation of endolysosomal cholesterol homeostasis can also impair viral infection. For example, itraconazole and fluoxetine induce endolysosomal cholesterol imbalance and inhibit Ebola virus infection *in vitro*, although their relevant molecular mechanisms are not identical (Kummer et al., 2022). Lipid-targeting effects should therefore be assigned to the experimentally demonstrated host target and viral stage rather than collectively labelled as NPC1 inhibition. This distinction is particularly important for itraconazole, which inhibits enterovirus RNA replication through oxysterol-binding protein (OSBP) and ORP4 rather than through an entry-stage NPC1 mechanism (Strating et al., 2015). Together, these studies illustrate that NPC1, OSBP/ORP-family lipid transport and related cholesterol-remodeling pathways represent distinct host vulnerabilities whose therapeutic relevance depends on the virus and the stage of the viral life cycle being targeted.

### 9.4 Translational prioritization

Collectively, the available evidence suggests several host-directed strategies that may offer greater translational tractability than sustained global CME inhibition. First, virus-specific receptor-adaptor or receptor-cofactor interfaces may provide greater selectivity than targeting constitutive clathrin assembly. Second, temporally restricted interventions directed at the short entry window may reduce host toxicity by limiting the duration of pathway perturbation. Third, downstream endosomal vulnerabilities-including lipid remodeling, ion-channel-dependent trafficking, protease activation and fusion competence-may be pharmacologically accessible even when the initial uptake route is heterogeneous. These approaches should be regarded as proposed translational priorities rather than established therapeutic solutions. Their advancement requires target-engagement studies, pharmacokinetic exposure compatible with antiviral concentrations, disease-relevant primary or organoid models, *in vivo* validation and explicit assessment of effects on essential host trafficking.

## 10 Discussion and perspectives

The evidence synthesized in this review supports a reframed interpretation of CME in viral entry. CME can provide a productive route for internalizing virus-receptor complexes and delivering them to endosomal compartments through clathrin/adaptor-mediated cargo recruitment, coat assembly, dynamin-mediated vesicle scission and Hsc70/auxilin-associated coat uncoating, thereby creating conditions for downstream low-pH triggering, proteolytic cleavage, membrane fusion, penetration or viral uncoating (Barouch et al., 1994; Fotin et al., 2004a; Mercer et al., 2010; McMahon and Boucrot, 2011; Ferguson and De Camilli, 2012; Sousa et al., 2016; Kaksonen and Roux, 2018). However, its contribution is conditional rather than universal. The strongest support remains concentrated in defined model systems, including VSV, SFV and HRV2/LDLR-mediated uptake, whereas evidence for DENV, HCV, JCPyV, hantaviruses, HCoV-229E, SARS-CoV-2 and several other viruses is more cell-, receptor-, protease- and assay-dependent. Consequently, claims of CME use should specify viral strain, receptor or cofactor usage, host-cell model, inoculum and assay window.

The operational categories proposed here are intended to standardize mechanistic wording rather than to function as a formal risk-of-bias scale. Their interpretation should remain explicitly linked to the viral strain or particle system, receptor/cofactor context, host-cell model and entry stage examined.

The remaining controversies center on pathway plasticity, cargo-specific adaptor use and translational specificity. Many viruses can shift among CME, macropinocytosis, lipid-raft/caveolae-associated endocytosis, clathrin-independent dynamin-dependent uptake and plasma-membrane fusion, particularly when receptor usage, cell type or experimental conditions change (Mercer et al., 2010; Grove and Marsh, 2011; Rennick et al., 2021). It is also unresolved how different viruses trigger or bypass *de novo* CCP formation, how particle size (Cureton et al., 2010), membrane tension (Boulant et al., 2011), local PI(4,5)P2/lipid composition (Antonescu et al., 2011), and alternative adaptors (Mukhopadhyay and Riezman, 2007; Schmid and McMahon, 2007; Chen and Zhuang, 2008; Traub and Bonifacino, 2013) affect CCP maturation (Loerke et al., 2009; Cocucci et al., 2012), and how these processes can be resolved during viral entry using single-particle tracking (Lakadamyali et al., 2003; Liu et al., 2020) and advanced fluorescence/super-resolution imaging (Li et al., 2015).

Future work should therefore define the exact entry stage and cellular context being tested. Single-particle tracking (Lakadamyali et al., 2003; Liu et al., 2020), TIRFM, advanced fluorescence/super-resolution imaging (Li et al., 2015), CLEM (Plitzko et al., 2009) and cryo-ET can connect receptor binding to CCP recruitment, vesicle scission, endosomal trafficking and fusion/uncoating, whereas spatial proteomics (Lundberg and Borner, 2019) may help identify host factors and subcellular neighborhoods associated with virus entry. Inducible degradation, short-term knockdown, CRISPR screening and rescue experiments can identify virus-specific CME-related dependencies while reducing adaptation artifacts. For drug development, chemical proteomics, target identification, selectivity profiling, short-window dosing and tissue- or cell-targeted delivery should be used to move CME-associated modulators from mechanistic probes toward realistic antiviral candidates (Margarucci et al., 2011; von Kleist et al., 2011; Cojocaru et al., 2020; Zhang et al., 2025).

## 11 Conclusions

CME is best interpreted as a stage-resolved and context-dependent host-entry platform rather than as a fixed pathway label. Mechanistic claims should distinguish association from involvement and strict dependence and should identify the specific entry checkpoint demonstrated in the relevant virus-receptor-cell system.

From a therapeutic perspective, sustained global CME blockade is unlikely to provide sufficient selectivity for most applications. More tractable opportunities may lie in virus-specific receptor-adaptor interactions and in downstream membrane, lipid, ion-channel and endosomal vulnerabilities that can be targeted within a defined entry window. Applying stage-resolved evidence standards to these targets may improve both mechanistic rigor and the prioritization of host-directed antiviral strategies.
