# Supplementary material for: Reframing clathrin-mediated viral entry: evidence standards, context dependence and host-directed vulnerabilities
Source: Front Cell Infect Microbiol. 2026 Oct 5;16:1929024. doi: 10.3389/fcimb.2026.1929024 (PMC13678436; doi:10.3389/fcimb.2026.1929024)
Supplement: Supplementary file 1 [file Table1.docx]

**Supplementary Table S1. Database-specific search strategy, selection criteria and evidence-prioritization logic**

Scope: This table documents the search strategy and evidence-prioritization logic used for a structured narrative review. Searches were designed to identify and triangulate mechanistically relevant evidence rather than to generate a PRISMA-style quantitative flow diagram or a prevalence estimate of CME usage among viruses. The database framework comprised PubMed and Web of Science Core Collection, supplemented by targeted virus-specific/tool-specific searches and citation tracking. The prespecified primary literature cutoff remains 18 June 2026. This targeted update should not be interpreted as a new systematic-search denominator.

**A. Database-specific search strategy**

| **Database** | **Exact search string / search approach** | **Date range, fields and limits** | **Supplementary retrieval and citation checking** |
| --- | --- | --- | --- |
| **PubMed** | (("clathrin-mediated endocytosis"[Title/Abstract] OR "clathrin-dependent endocytosis"[Title/Abstract] OR "clathrin-coated pit"[Title/Abstract] OR "clathrin-coated pits"[Title/Abstract] OR "clathrin-coated vesicle"[Title/Abstract] OR "clathrin-coated vesicles"[Title/Abstract] OR "clathrin heavy chain"[Title/Abstract] OR CLTC[Title/Abstract] OR "adaptor protein complex 2"[Title/Abstract] OR "AP-2"[Title/Abstract] OR "AP-2 adaptor"[Title/Abstract] OR "AP2 adaptor"[Title/Abstract] OR "AP2 complex"[Title/Abstract] OR epsin[Title/Abstract] OR Eps15[Title/Abstract] OR CALM[Title/Abstract] OR AP180[Title/Abstract] OR dynamin[Title/Abstract] OR AAK1[Title/Abstract] OR GAK[Title/Abstract] OR auxilin[Title/Abstract] OR Hsc70[Title/Abstract] OR HSPA8[Title/Abstract]) AND ("viral entry"[Title/Abstract] OR "virus entry"[Title/Abstract] OR internalization[Title/Abstract] OR endocytosis[Title/Abstract] OR penetration[Title/Abstract] OR fusion[Title/Abstract] OR uncoating[Title/Abstract] OR "genome release"[Title/Abstract] OR infection[Title/Abstract] OR inhibitor[Title/Abstract] OR "small molecule"[Title/Abstract] OR peptide[Title/Abstract] OR "natural product"[Title/Abstract] OR "host-directed antiviral"[Title/Abstract])) AND ("1900/01/01"[Date - Publication] : "2026/06/18"[Date - Publication]) | Database inception to 18 June 2026. Publication-date upper limit set to 18 June 2026. Main query restricted to Title/Abstract fields. No language restriction was applied at the search stage. Records were screened for mechanistic relevance rather than by automated document-type filters. | Targeted supplementary retrieval combined individual virus names, receptor names, CME machinery terms and compound/tool names with clathrin, clathrin-coated pit, CME, AP-2/AP2 adaptor, dynamin, endocytosis, viral entry, fusion, penetration, uncoating, genome release or infection terms. Backward and forward citation tracking of key mechanistic studies and authoritative reviews was used to identify older foundational papers, methodological papers and tool-specific reports. |
| **Web of Science Core Collection** | TS=("clathrin-mediated endocytosis" OR "clathrin-dependent endocytosis" OR "clathrin-coated pit" OR "clathrin-coated pits" OR "clathrin-coated vesicle" OR "clathrin-coated vesicles" OR "clathrin heavy chain" OR CLTC OR "adaptor protein complex 2" OR "AP-2" OR "AP-2 adaptor" OR "AP2 adaptor" OR "AP2 complex" OR epsin OR Eps15 OR CALM OR AP180 OR dynamin OR AAK1 OR GAK OR auxilin OR Hsc70 OR HSPA8) AND TS=("viral entry" OR "virus entry" OR internalization OR endocytosis OR penetration OR fusion OR uncoating OR "genome release" OR infection OR inhibitor OR "small molecule" OR peptide OR "natural product" OR "host-directed antiviral") | Database inception to 18 June 2026. Topic Search (TS) was used. Records published after the final literature date were excluded during screening when date filtering was not available in the search interface. No language restriction was applied at the search stage. Document types were screened for mechanistic relevance rather than used as strict automated exclusion filters. | Targeted supplementary retrieval combined individual virus names, receptor names, CME machinery terms and compound/tool names with clathrin, clathrin-coated pit, CME, AP-2/AP2 adaptor, dynamin, endocytosis, viral entry, fusion, penetration, uncoating, genome release or infection terms. Backward and forward citation tracking of key mechanistic studies and authoritative reviews was used to identify older foundational papers, methodological papers and tool-specific reports. |

**B. Record-management and count-use policy**

| **Component** | **Implementation in this review** | **Interpretation** |
| --- | --- | --- |
| **Database output** | PubMed and Web of Science Core Collection searches provided the main retrieval set for mechanistic screening. Targeted searches and citation tracking were used to capture virus-specific, receptor-specific, cell-model-specific and tool-specific studies that might not be retrieved by the broad database query. | Search outputs were used to locate relevant mechanistic evidence, not to estimate the proportion of viruses using CME. |
| **Deduplication and screening** | Records from databases, targeted searches and citation tracking were screened for mechanistic relevance, stage resolution, virus-receptor-cell context and relevance to the main text, Table 2 and Table 3. | A PRISMA-style retrieved/screened/included flow was not constructed because the review was not designed as a systematic review or meta-analysis. Representative table entries should not be interpreted as a deduplicated flow denominator. |
| **Evidence inclusion** | Original experimental studies were prioritized. Mechanistic CME studies and selected authoritative reviews were included when they supported interpretation of viral-entry experiments, experimental perturbation tools or translational limitations. | Inclusion reflects evidence relevance and interpretive value rather than a numerical prevalence inventory. |

**C. Selection, exclusion and evidence-prioritization logic**

| **Item** | **Selection logic** | **Exclusion or downgrading logic** | **Use in the review** |
| --- | --- | --- | --- |
| **General inclusion criteria** | Prioritized original experimental studies that directly examined viral attachment, receptor engagement, internalization, clathrin-associated uptake, dynamin-dependent scission, endosomal delivery, fusion, penetration, capsid remodeling, viral uncoating, genome release, internalized input-genome detection or assays specifically identifying newly synthesized viral RNA or early productive infection in relation to CME or CME-associated pathways. Mechanistic CME studies and selected authoritative reviews were included when they informed interpretation of viral-entry experiments. | Strict CME-dependent entry was not assigned from endpoint infection reduction, low-pH sensitivity, dynamin dependence, broad endosomal perturbation, single non-specific inhibitor sensitivity or static colocalization without stage-resolved entry evidence. | Evidence-prioritized narrative inclusion; not a quantitative prevalence estimate of CME usage among viruses. |
| **Viruses summarized in Table 2** | Included viruses when experimental evidence supported involvement of CME, CME-associated uptake, clathrin-associated endosomal delivery or related early entry events in human cells, disease-relevant mammalian models or well-established viral-entry model systems. | Classified conservatively when evidence was insufficiently defined by virus strain, receptor/cofactor usage, host-cell model, assay window or affected entry stage. Serotype-level, family-level or single-cell-model findings were not extrapolated to universal CME dependence. | Representative virus entries prioritized by human relevance, model relevance and mechanistic evidence strength. |
| **Experimental tools summarized in Supplementary Table S3** | Included tools commonly used to interrogate clathrin-coated-pit formation, adaptor recruitment, cargo selection, dynamin-dependent scission, coat uncoating, AAK1/GAK-regulated trafficking, endosomal acidification, endosomal maturation or trafficking steps relevant to viral entry. | Compounds were not treated as direct CME inhibitors when they primarily affected dynamin, endosomal pH, PIKfyve-dependent trafficking, broad membrane trafficking, kinase networks or viability. Natural products and derivatives were interpreted as chemical probes or model-specific leads unless target engagement, selectivity, reversibility and in vivo efficacy were established. | Representative entry-modulation tools prioritized by experimental use and relevance to CME-associated viral-entry interpretation. |
| **Host-directed strategies summarized in Table 3** | Included host-directed strategies when experimental or translational evidence supported modulation of CME-associated viral entry or related host pathways, with the affected host target, viral stage, virus-receptor-cell context and model relevance sufficiently defined. | Strategies were downgraded when antiviral activity was inferred mainly from endpoint infection reduction, broad cytotoxicity or nonspecific trafficking perturbation without defined target engagement, entry-stage linkage or adequate controls. Preclinical activity was not extrapolated to clinical efficacy without corresponding translational evidence. | Representative host-directed strategies prioritized by mechanistic specificity, model relevance, breadth of antiviral evidence and translational support. |
| **Evidence grading and synthesis** | Used stage-resolved criteria. CME-associated entry denotes limited or hypothesis-generating evidence. CME-involved entry requires concordant evidence from at least two independent approaches, including at least one CME-proximal perturbation or dynamic imaging approach, linked to an entry-window functional effect. CME-dependent entry is reserved for defined virus-receptor-cell contexts in which direct perturbation of core/proximal CME machinery with rescue or orthogonal validation is linked to productive progression through clathrin/AP-2-positive CCPs/CCVs and an early functional entry readout. | Evidence is downgraded when viability, receptor expression, viral binding, representative CME cargo uptake, alternative endocytic routes or post-entry effects are not adequately controlled. Pharmacological evidence is supportive rather than universally mandatory when stronger direct mechanistic evidence is available. | Mechanistic classification and evidence confidence are reported separately and remain context specific. |

**Notes:** This review was designed as a structured narrative review rather than a systematic review or meta-analysis. No formal risk-of-bias assessment, PRISMA flow diagram or quantitative meta-analysis was undertaken. The synthesis should be interpreted as an evidence-graded mechanistic review rather than a prevalence estimate of CME usage among viruses. Database record counts were not used as inclusion metrics and were not retrospectively reconstructed as systematic-review denominators.

**Abbreviations:** AAK1, adaptor-associated kinase 1; AP-2, adaptor protein complex 2; AP180, adaptor protein 180; CALM, clathrin assembly lymphoid myeloid leukemia protein; CCV, clathrin-coated vesicle; CCP, clathrin-coated pit; CME, clathrin-mediated endocytosis; GAK, cyclin G-associated kinase; Hsc70, heat shock cognate 70; HSPA8, heat shock protein family A member 8; PIKfyve, phosphoinositide kinase, FYVE-type zinc finger containing.
