# Supplementary material for: Reframing clathrin-mediated viral entry: evidence standards, context dependence and host-directed vulnerabilities
Source: Front Cell Infect Microbiol. 2026 Oct 5;16:1929024. doi: 10.3389/fcimb.2026.1929024 (PMC13678436; doi:10.3389/fcimb.2026.1929024)
Supplement: Supplementary file 2 [file Table2.docx]

**Supplementary Table S2. Evidence matrix and independent coding of CME-related viral-entry claims**

**Purpose:** This supplementary matrix provides the detailed evidence basis underlying the P/G/I/F designations in main-text Table 2. Two reviewers (QW and YX) independently evaluated the mechanistic classification of each virus/model using the prespecified evidence framework before comparison of their assessments. Disagreements were resolved by consensus, with third-author adjudication when required. The study-level evidence basis and final adjudicated classifications are reported below.

**A. Evidence basis supporting the main-text P/G/I/F designations**

| **Virus/model** | **Key supporting studies** | **Particle system / cell model** | **Receptor/cofactor role** | **Evidence domains**  **(P/G/I/F)** | **Evidence basis / principal findings** | **Entry stage resolved** | **Alternative route / key caveat** | **Preliminary mechanistic category** | **Preliminary confidence** |
| --- | --- | --- | --- | --- | --- | --- | --- | --- | --- |
| Human immunodeficiency virus type 1 (HIV-1) | Daecke et al., 2005; Miyauchi et al., 2009; Gobeil et al., 2013; Herold et al., 2014; Li et al., 2017 | HeLa-derived CD4+ cells, HeLaP4, 293T, TZM-bl and Rev-CEM. Limit conclusions to the tested strain, receptor/coreceptor usage and activation state. | CD4 with CCR5 or CXCR4; receptor/coreceptor usage determines tropism. | P△ / G△ / I✓ / F✓ | CME-associated uptake has been reported in several engineered or permissive systems; however, productive HIV-1 entry frequently occurs by plasma-membrane fusion and can also involve macropinocytosis or lipid-raft-associated uptake. | Internalization/fusion; productive entry route remains disputed. | Plasma-membrane fusion, macropinocytosis and other routes. | CME-associated | Moderate, context-dependent |
| Vesicular stomatitis virus (VSV; zoonotic/model virus) | Matlin et al., 1982; Le Blanc et al., 2005; Cureton et al., 2009, 2010; Johannsdottir et al., 2009; Finkelshtein et al., 2013 | HeLa, MDCK, BS-C-1 and related epithelial models. AP-2 and actin requirements can vary with model and particle geometry. | LDLR and LDLR-family members. | P✓ / G✓ / I✓ / F✓ | Classical stage-supported CME model: virions can induce de novo clathrin-coat nucleation at binding sites and enter through clathrin-coated structures; incompletely coated carriers may require actin. | CCP nucleation, internalization and endosomal delivery. | AP-2 and actin requirements can vary; do not generalize to all rhabdoviruses or cell types. | CME-dependent | High, model-defined |
| Semliki Forest virus (SFV; historical/model alphavirus) | Helenius et al., 1980; Marsh et al., 1983; Clark et al., 2022; Cao et al., 2023; Yang et al., 2024 | BHK-21 and related permissive models. Historical/model evidence should not be generalized to all human target cells. | VLDLR and ApoER2 are established entry receptors in defined SFV models. | P△ / G✓ / I✓ / F✓ | Historical studies support productive clathrin-associated uptake and low-pH endosomal entry; receptor studies identify VLDLR/ApoER2, and structural work defines receptor-binding modes. Virions traffic through early endosomal compartments in classical models. | Internalization and early endosomal delivery/fusion. | Historical/model systems; receptor and host-cell context matter. | CME-dependent | High, model-defined |
| Sindbis virus (SINV) | Abell and Brown, 1993; DeTulleo and Kirchhausen, 1998; Glomb-Reinmund and Kielian, 1998; Smit et al., 1999 | HeLa, BHK-21 and related permissive cells. Treat as endosomal entry unless stronger CME-specific stage-resolved evidence is available. | GAGs and cell-type-specific attachment/entry factors. | P△ / G– / I△ / F✓ | Low-pH-triggered entry and fusion are supported, whereas direct stage-resolved evidence for strict CME-dependent entry in defined virus-receptor-cell systems remains limited. | Low-pH-triggered endosomal entry and fusion. | Direct CME-specific and stage-resolved evidence is limited in defined virus-receptor-cell systems. | CME-associated | Low, hypothesis-generating |
| Dengue virus (DENV) | van der Schaar et al., 2008; Acosta et al., 2009; Peng et al., 2009; Suksanpaisan et al., 2009; Cruz-Oliveira et al., 2015; Piccini et al., 2015; Ho et al., 2017 | Vero, HepG2 and other mammalian cells. Route usage varies by serotype and cell context. | GAGs, DC-SIGN and other cell-type-specific attachment/entry factors. | P✓ / G✓ / I✓ / F✓ | CME has been reported for several DENV serotypes and cell systems; however, clathrin-independent, dynamin-dependent entry has also been demonstrated. | Internalization and fusion/endosomal entry. | Clathrin-independent, dynamin-dependent uptake occurs in selected systems; serotype alone is insufficient to infer CME dependence. | CME-involved | Moderate, context-dependent |
| Hepatitis C virus (HCV) | Blanchard et al., 2006; Meertens et al., 2006; Helle and Dubuisson, 2008; Inoue et al., 2018 | Huh7/Huh7.5 and derivative hepatoma cells. Specify HCVcc, HCVpp or other viral systems. | HSPGs/GAGs, LDLR, SR-BI, CD81, CLDN1, OCLN and additional entry cofactors. | P✓ / G✓ / I△ / F✓ | CME-associated entry has been reported in HCVcc/HCVpp and hepatoma-cell systems, but apparent dependence varies with viral system, receptor/cofactor expression and assay design. | Internalization and early endosomal delivery. | Apparent dependence varies with particle system, receptor/cofactor expression and assay design. | CME-involved | Moderate, context-dependent |
| Human rhinovirus 2 (HRV2; minor group) | Bayer et al., 2001; Snyers et al., 2003; Brabec-Zaruba et al., 2009 | HeLa and related susceptible cells. Interpretation is specific to minor-group HRV2/LDLR. | LDLR-family receptors. | P✓ / G✓ / I✓ / F✓ | Well-characterized minor-group rhinovirus using LDLR-mediated receptor endocytosis with clathrin-associated uptake and endosomal uncoating. | Internalization and endosomal uncoating. | Specific to minor-group HRV2; do not generalize to all rhinoviruses. | CME-dependent | High, model-defined |
| Mammalian orthoreovirus (MRV; human-associated isolates) | Barton et al., 2001; Maginnis et al., 2006, 2008; Schulz et al., 2012; Koehler et al., 2021 | BS-C-1, L929, GCF and related models. Specify strain/isolate origin and avoid broad extrapolation. | Sialylated glycans/JAM-A support attachment; β1 integrin primarily supports post-attachment internalization and clathrin recruitment. | P△ / G✓ / I✓ / F✓ | Following attachment through JAM-A and strain-dependent glycan interactions, β1 integrin promotes internalization. NPXY motifs in the β1-integrin cytoplasmic tail contribute to functional entry, and direct integrin engagement can recruit clathrin. | Internalization, endosomal trafficking and proteolysis. | Multiple endocytic routes remain possible depending on strain and cell context. | CME-involved | Moderate-to-high, model-defined |
| Influenza A virus (IAV) | Matlin et al., 1981; Sieczkarski and Whittaker, 2002; Rust et al., 2004; Sun et al., 2017; Du et al., 2024 | HeLa, MDCK, BS-C-1 and other epithelial cells. Route usage depends on strain, cell type and multiplicity. | Sialic-acid-containing glycans; receptor/adaptor context can include epsin-1. | P✓ / G✓ / I✓ / F✓ | Many particles following a clathrin-associated route induce virus-centered CCPs; epsin-1 and virus-induced CCP nucleation are important in defined systems, while alternative entry pathways remain available. | Virus-centered CCP formation, internalization and fusion. | Macropinocytosis and other routes are documented; CME is not obligatory or exclusive. | CME-involved | High, model-defined |
| JC polyomavirus (JCPyV) | Pho et al., 2000; Elphick et al., 2004; Querbes et al., 2004, 2006; Neu et al., 2010; Assetta et al., 2013; Mayberry et al., 2019 | HeLa-H1, SVG-A and glial-lineage models. Distinguish JCPyV from other polyomaviruses. | LSTc-containing sialylated glycans; 5-HT2R and β-arrestin-linked entry cofactors. | P✓ / G✓ / I✓ / F✓ | Clathrin-associated or CME-involved uptake has been reported in defined cell systems, but JCPyV entry is strongly receptor- and cell-type-dependent. | Internalization and downstream endosomal sorting. | Receptor/cell context matters; caveolar sorting can occur downstream. | CME-involved | Moderate-to-high, model-defined |
| Hantaan virus (HTNV) | Gavrilovskaya et al., 1999; Jin et al., 2002; Mou et al., 2006; Gu et al., 2024; Ma et al., 2024 | Vero E6, A549, Jurkat T and related models. Explicitly distinguish authentic-virus from pseudotyped-particle studies. | Reported factors include αvβ3 integrin, TIM-family phosphatidylserine receptors and other candidate host factors. | P△ / G△ / I△ / F✓ | Clathrin-associated and dynamin-sensitive entry has been reported, but receptor evidence is heterogeneous. TIM-family activity has been demonstrated in selected systems, whereas broader genetic studies question a universal requirement for proposed integrins. | Internalization/early infection. | Authentic versus pseudotyped systems and receptor controversy; genetic studies question universal integrin requirements. | CME-associated | Moderate, context-dependent |
| Crimean-Congo hemorrhagic fever virus (CCHFV) | Simon et al., 2009; Garrison et al., 2013; Shtanko et al., 2014; Xu et al., 2024 | Vero E6 and other permissive cells. Treat receptor and CME assignments as model-defined. | LDLR and other host factors have been implicated. | P✓ / G△ / I△ / F✓ | Early infection is sensitive to cholesterol and endocytic-trafficking perturbation; CME-associated entry has been reported, but direct clathrin/AP-2 stage-resolved evidence remains limited. | Early endosomal entry/trafficking and multivesicular-body progression. | Evidence relies substantially on endocytic/cholesterol perturbation and permissive cell models; direct CME-specific evidence is limited. | CME-associated | Low-to-moderate, context-dependent |
| Borna disease virus 1 (BoDV-1/BDV) | Clemente and de la Torre, 2009 | OL, Vero E6, Huh-7 and neuronal/glial models. Receptor identity remains unresolved. | Host receptor not definitively established; viral glycoprotein G mediates attachment. | P✓ / G✓ / I△ / F✓ | Experimental evidence supports a clathrin-associated, caveola-independent endocytic entry pathway in the tested systems; microtubules contribute mainly to post-entry transport. | Internalization and early endosomal trafficking; microtubule-dependent post-entry transport. | Host receptor is unresolved and evidence is model-limited. | CME-involved | Moderate, context-dependent |
| SARS-CoV-2 | Hoffmann et al., 2020; Bayati et al., 2021; Koch et al., 2021 | ACE2-expressing epithelial systems; pseudotype/authentic-virus and protease context must be specified. | ACE2; TMPRSS2 and cathepsins influence surface-fusion versus endosomal entry. | P✓ / G✓ / I△ / F✓ | Spike-ACE2 uptake and spike-pseudotyped virus infection support clathrin-associated uptake in some systems, but productive entry can occur through TMPRSS2-driven surface fusion or cathepsin-dependent endosomal routes. | Internalization/endosomal entry versus plasma-membrane fusion. | TMPRSS2-dependent surface fusion; spike-ACE2 uptake should not be equated with productive CME infection. | CME-associated | Moderate, protease/cell-context-dependent |
| Human coronavirus 229E (HCoV-229E) | Kawase et al., 2009; Bertram et al., 2013; Shirato et al., 2018; Andreu et al., 2024 | Evidence is mainly from Huh-7 cells; respiratory epithelial models and protease-dependent route selection require separate interpretation. | Aminopeptidase N (APN/CD13) is the canonical receptor. | P✓ / G✓ / I△ / F✓ | A Huh-7 cell study reports reduced HCoV-229E entry/infection after CME-perturbing tools and AP-2 μ-subunit knockout, supporting CME involvement as one possible entry route in Huh-7 cells. | Internalization/endosomal entry in Huh-7 cells. | Respiratory epithelial models may favor TMPRSS2-dependent entry; evidence is model-limited. | CME-involved | Moderate, model-limited |
| Zika virus (ZIKV) | Hamel et al., 2015; Hastings et al., 2017; Meertens et al., 2017; Persaud et al., 2018; Owczarek et al., 2019; Li et al., 2020 | Human neural-cell evidence is mainly derived from T98G cells; caveola-related perturbations also affect entry. | AXL, TIM/TAM factors, HSPGs and other cell-specific attachment/entry cofactors. | P✓ / G✓ / I△ / F✓ | In human glioblastoma T98G cells, ZIKV entry is reduced by chlorpromazine, Pitstop2 and clathrin heavy-chain siRNA, supporting CME involvement. | Internalization/early infection in T98G cells. | Caveola-related and other routes may contribute; disease-relevant neural-cell evidence remains model-limited. | CME-involved | Moderate, context-dependent |
| West Nile virus (WNV) | Chu and Ng, 2004a, 2004b; Davis et al., 2006 | Permissive mammalian models; primary human target-cell validation remains limited. | HSPGs, DC-SIGN/DC-SIGNR, αvβ3 integrin and other reported cell-dependent factors. | P✓ / G✓ / I✓ / F✓ | Dominant-negative Eps15, clathrin-pathway inhibitors and immunoelectron microscopy show WNV particles in clathrin-coated pits and reduced infectious entry. | CCP association/internalization and infectious entry. | Strong permissive-model evidence but limited primary human target-cell validation. | CME-involved | High, model-defined |
| Japanese encephalitis virus (JEV) | Nawa et al., 2003; Kalia et al., 2013; Liu et al., 2017; Yun and Lee, 2018 | Vero/BHK-21 versus neuronal/fibroblast models; conclusions should remain cell-type specific. | HSPGs, laminin receptor, integrins and other cell-context-dependent cofactors. | P✓ / G✓ / I△ / F✓ | CME-associated entry has been reported in Vero and BHK-21 cells, including clathrin/dynamin/Rab5/Rab11 dependence in BHK-21 cells. | Internalization/endosomal trafficking. | Clathrin-independent entry is reported in selected neuronal/fibroblast models. | CME-involved | Moderate, cell-context-dependent |
| Chikungunya virus (CHIKV) | Bernard et al., 2010; Hoornweg et al., 2016; Zhang et al., 2018; Lee et al., 2019; Feng et al., 2023 | Mammalian live-cell imaging models; strain, receptor usage and host-cell context matter. | MXRA8, GAGs and other attachment/entry cofactors. | P✓ / G△ / I✓ / F✓ | Live single-virus tracking shows many fusion-competent particles colocalize with clathrin before fusion; receptor studies support alphavirus internalization after receptor engagement. | Internalization and membrane fusion. | Macropinocytosis/clathrin-independent entry is reported in some host cells. | CME-involved | Moderate, context-dependent |
| Human adenovirus type 2/5 (HAdV-2/5) | Wickham et al., 1993; Bergelson et al., 1997; Wang et al., 1998; Meier et al., 2002 | Human epithelial, HeLa and HEK models. Distinguish CAR-mediated attachment from integrin-driven internalization. | CAR mediates attachment; αv integrins promote internalization. | P△ / G✓ / I✓ / F✓ | HAdV-2 uptake into epithelial cells is inhibited by dominant-negative clathrin hub/Eps15 and dynamin K44A, linking clathrin-associated uptake to early productive entry after integrin engagement. | Internalization and endosomal escape. | Macropinocytosis can accompany uptake; attachment and internalization receptor roles differ. | CME-involved | Moderate, context-dependent |
| Enterovirus A71 (EV-A71) | Hussain et al., 2011; Lin et al., 2013; Chen et al., 2019; Zhu et al., 2023 | Human rhabdomyosarcoma and other susceptible cells. Alternative ARF6/endophilin/caveolar routes occur in specific models. | SCARB2, PSGL-1 and other cell-type-dependent attachment/entry factors. | P✓ / G✓ / I✓ / F✓ | Targeted siRNA screening, dominant-negative Eps15, clathrin colocalization, TEM and inhibitor assays identify CME genes, including AP2A1 and CHC, as required for infectious entry. | Internalization/infectious entry. | ARF6/endophilin/caveolar routes are reported in specific cell types. | CME-involved | Moderate-to-high, context-dependent |
| Rabies virus (RABV) | Lafon, 2005; Piccinotti et al., 2013; Wang et al., 2019, 2023; Sun et al., 2025 | BS-C-1, HEK293, N2a and primary neuronal models. Human neuronal validation remains comparatively limited. | nAChR, NCAM, p75NTR, TfR1, NRP2 and other reported neuronal or epithelial entry factors. | P✓ / G✓ / I✓ / F✓ | RABV uptake is predominantly clathrin-associated and dynamin-sensitive in epithelial and neuronal models; AAK1/AP2M1 phosphorylation and TfR1-coupled endocytosis support a CME-linked entry mechanism. | Internalization and early endosomal entry. | Human neuronal validation remains limited; receptor usage is model-dependent. | CME-involved | Moderate, context-dependent |

**Evidence-domain legend:** P, pharmacological evidence; G, genetic evidence; I, imaging evidence; F, stage-resolved functional evidence. ✓, direct or sufficiently convergent support; △, partial, indirect or context-limited support; –, not demonstrated or not adequately assessed. The symbols are descriptive rather than numerical and should not be summed to infer CME dependence.

**B. Independent author coding and adjudication**

| **Virus/model** | **Reviewer A classification** | **Reviewer B classification** | **Disagreement?** | **Final adjudication / rationale** |
| --- | --- | --- | --- | --- |
| Human immunodeficiency virus type 1 (HIV-1) | CME-associated | CME-associated | Agreement | CME-associated |
| Vesicular stomatitis virus (VSV; zoonotic/model virus) | CME-dependent | CME-dependent | Agreement | CME-dependent |
| Semliki Forest virus (SFV; historical/model alphavirus) | CME-dependent | CME-dependent | Agreement | CME-dependent |
| Sindbis virus (SINV) | CME-associated | CME-associated | Agreement | CME-associated |
| Dengue virus (DENV) | CME-involved | CME-involved | Agreement | CME-involved |
| Hepatitis C virus (HCV) | CME-involved | CME-involved | Agreement | CME-involved |
| Human rhinovirus 2 (HRV2; minor group) | CME-dependent | CME-dependent | Agreement | CME-dependent |
| Mammalian orthoreovirus (MRV; human-associated isolates) | CME-involved | CME-involved | Agreement | CME-involved |
| Influenza A virus (IAV) | CME-involved | CME-involved | Agreement | CME-involved |
| JC polyomavirus (JCPyV) | CME-involved | CME-involved | Agreement | CME-involved |
| Hantaan virus (HTNV) | CME-associated | CME-associated | Agreement | CME-associated |
| Crimean-Congo hemorrhagic fever virus (CCHFV) | CME-associated | CME-associated | Agreement | CME-associated |
| Borna disease virus 1 (BoDV-1/BDV) | CME-involved | CME-involved | Agreement | CME-involved |
| SARS-CoV-2 | CME-associated | CME-associated | Agreement | CME-associated |
| Human coronavirus 229E (HCoV-229E) | CME-involved | CME-involved | Agreement | CME-involved |
| Zika virus (ZIKV) | CME-involved | CME-involved | Agreement | CME-involved |
| West Nile virus (WNV) | CME-involved | CME-involved | Agreement | CME-involved |
| Japanese encephalitis virus (JEV) | CME-involved | CME-involved | Agreement | CME-involved |
| Chikungunya virus (CHIKV) | CME-involved | CME-involved | Agreement | CME-involved |
| Human adenovirus type 2/5 (HAdV-2/5) | CME-involved | CME-involved | Agreement | CME-involved |
| Enterovirus A71 (EV-A71) | CME-involved | CME-dependent | Disagreement | CME-involved. Multiple orthogonal evidence domains support CME-mediated EV-A71 entry, but alternative ARF6-, endophilin- and caveola-associated routes in specific cell models preclude assignment of universal CME dependence. |
| Rabies virus (RABV) | CME-involved | CME-involved | Agreement | CME-involved |

**Author-coding note:** Reviewer A and Reviewer B completed their classifications independently before comparison. Initial agreement was observed for 21 of 22 virus/model classifications (95.5%). One disagreement, involving EV-A71, was resolved during adjudication, with CME-involved retained as the final classification.Initial agreement was 95.5% (21/22), with a Cohen’s κ of 0.917.
