# Supplementary material for: Reframing clathrin-mediated viral entry: evidence standards, context dependence and host-directed vulnerabilities
Source: Front Cell Infect Microbiol. 2026 Oct 5;16:1929024. doi: 10.3389/fcimb.2026.1929024 (PMC13678436; doi:10.3389/fcimb.2026.1929024)
Supplement: Supplementary file 3 [file Table3.docx]

**Supplementary Table S3. Experimental tools used to interrogate CME and CME-associated viral-entry checkpoints**

Values are separated according to their experimental meaning. Working concentrations, biochemical IC50 values and cellular potency values should not be compared directly. Each value is reported together with the corresponding assay, cell model or experimental system, exposure conditions where applicable, and major interpretive limitations.

| **Category** | **Compound/tool** | **Primary target or step** | **Value type** | **Reported value** | **Assay/cell model/exposure** | **Interpretive limitation** | **Reference** |
| --- | --- | --- | --- | --- | --- | --- | --- |
| CME-proximal | Pitstop 1 | Clathrin N-terminal-domain ligand interactions | Biochemical PPI IC50 | 18 µM | Assay: ELISA inhibition of recombinant clathrin NTD-amphiphysin-1 B/C-domain binding. Cell model: cell-free recombinant proteins. Exposure: no routine extracellular live-cell assay because of poor membrane penetration; intracellular validation used microinjection into lamprey reticulospinal axons followed by 5-Hz stimulation for 20 min. | The 18 µM value is a biochemical PPI IC50, not a cellular CME IC50; poor membrane penetration limits bath application. | von Kleist et al., 2011 |
| CME-proximal | Pitstop 2 | Clathrin N-terminal-domain ligand interactions / CME | Cellular CME IC50 | 12-15 µM (Tf uptake) | Assay: Alexa568-transferrin uptake. Cell model: HeLa cells. Exposure: 15-min preincubation, then 15-min Tf internalization at 37 °C in the continued presence of inhibitor; 30 µM produced near-complete inhibition. | Not CME-selective in cells: later work showed inhibition of clathrin-independent cargo uptake; use orthogonal/genetic controls. | von Kleist et al., 2011; Dutta et al., 2012 |
| CME-proximal | Tyrphostin A23 | AP-2 µ2 interaction with tyrosine motifs | Working concentration | 10-200 µM | Transferrin-receptor internalization | Cargo-selective; not a universal CME inhibitor | Banbury et al., 2003 |
| CME-proximal | Chlorpromazine | Clathrin/AP-2 redistribution; membrane/trafficking effects | Working concentration | 5-30 µM | Historical CME-sensitive uptake/virus-entry assays | Broad cationic amphiphile; membrane and toxicity effects | Wang et al., 1993; Daniel et al., 2015 |
| CME-proximal | WBox2 | Clathrin terminal-domain-derived peptide | Working concentration | 5, 10 and 20 µM for concentration-dependent CCP analyses; 55 µM in initial peptide screen | Transferrin uptake and dual-color TIRFM/cmeAnalysis; assay-dependent preincubation | Not an IC50; delivery/control peptide and cytotoxicity require validation | Chen et al., 2020 |
| Historical/delivery-limited | Hsc70/auxilin inhibitory peptides | Clathrin-coat uncoating | Working concentration in specialized models | ~3 µM in reported microinjection/neuronal contexts | Biochemical or neuronal microinjection/synaptic-vesicle systems | Not an established general cell-permeable viral-entry tool | Morgan et al., 2001; Sousa and Lafer, 2015 |
| Natural-product probe | Ikarugamycin | CME-associated internalization | Working concentration | 1-5 µM | Transferrin uptake and cell-dependent endocytosis assays | Target not fully resolved; dose/cell dependence | Elkin et al., 2016 |
| Dynamin | Dynasore | Dynamin GTPase / vesicle scission | Working concentration | 40-100 µM | Transferrin/receptor/virus internalization assays | Dynamin dependence is not CME specificity | Macia et al., 2006 |
| Dynamin | Dyngo-4a | Dynamin I/II helical GTPase; dynamin-dependent endocytosis | Biochemical DynI IC50; cellular CME IC50 | DynI: 0.38 ± 0.05 µM; CME: 5.7 ± 1.0 µM | Assay: PS-liposome-stimulated helical DynI GTPase (detergent-free) / automated Tfn-A594 uptake. Cell model: native sheep-brain DynI / U2OS human osteosarcoma cells. Exposure: U2OS cells preincubated 30 min; Tfn-A594 uptake for 8 min at 37 °C. | Biochemical and cellular IC50 values are assay-specific and not interchangeable; dynamin inhibition does not by itself establish CME specificity. | McCluskey et al., 2013; Basagiannis et al., 2021 |
| Dynamin | Dyngo-6a | Dynamin I helical GTPase; dynamin-dependent endocytosis | Biochemical DynI IC50; cellular CME IC50 | DynI: 3.2 ± 0.3 µM; CME: 5.8 ± 0.8 µM | Assay: PS-liposome-stimulated helical DynI GTPase (detergent-free) / automated Tfn-A594 uptake. Cell model: native sheep-brain DynI / U2OS human osteosarcoma cells. Exposure: U2OS cells preincubated 30 min; Tfn-A594 uptake for 8 min at 37 °C. | Assay-specific; dynamin-dependent uptake is not intrinsically CME-selective. Do not pool Dyngo-6a with Dyngo-4a as a family potency range. | McCluskey et al., 2013 |
| Dynamin | Dynole 2-24 | Dynamin I/II GTPase; dynamin-dependent CME | Biochemical DynI IC50; cellular CME IC50 | DynI IC50 0.56 ± 0.09 µM; CME IC50 1.9 ± 0.3 µM | In-vitro DynI GTPase assay; automated transferrin-uptake CME assay in U2OS human osteosarcoma cells; 30-min compound preincubation. | Assay-specific; cellular CME IC50 is not a biochemical DynII IC50. Do not merge with Dynole 34-2 as a family potency range. | Gordon et al., 2013 |
| Dynamin | Dynole 34-2 | Dynamin I GTPase; dynamin-dependent RME/CME | Biochemical DynI IC50; cellular RME IC50 | DynI IC50 1.30 ± 0.30 µM; RME IC50 5.0 ± 0.9 µM | PS-liposome-stimulated DynI GTPase (Malachite Green Pi-release; 30 min, 37 °C); U2OS transferrin uptake, 30-min preincubation + 8-min pulse at 37 °C. | Biochemical and cellular potencies are assay-specific; do not extrapolate to DynII or combine different dynoles into one range. | Hill et al., 2009 |
| Dynamin | MiTMAB | Dynamin-phospholipid interaction | Working concentration | 5-30 µM | Cellular endocytosis/cytokinesis assays | Membrane-active; off-target interpretation | Quan et al., 2007; Joshi et al., 2010 |
| Dynamin | OcTMAB | Dynamin-phospholipid interaction | Working concentration | 5-30 µM | Cellular endocytosis/cytokinesis assays | Membrane-active; off-target interpretation | Quan et al., 2007; Joshi et al., 2010 |
| Dynamin | Pthaladyn (23/29) | Dynamin I/II GTPase; GTP-competitive | Biochemical IC50; cellular SVE/RME | Pthaladyn-29: DynI IC50 4.58 ± 0.06 µM; DynII IC50 40.0 µM. Pthaladyn-23: DynI IC50 17.4 ± 5.8 µM; DynII IC50 63.1 µM; SVE IC50 12.9 ± 5.9 µM; RME inactive. | In-vitro DynI/DynII GTPase assays; transferrin endocytosis in U2OS cells; FM4-64 uptake in isolated rat-brain synaptosomes | Analogue- and assay-specific: Pthaladyn-29 was strongest biochemically, whereas Pthaladyn-23 was the only analogue with notable SVE inhibition in the original study. | Odell et al., 2010 |
| Dynamin | Rhodadyn (C10/D10) | Dynamin I GTPase / receptor-mediated endocytosis | Biochemical IC50; cellular RME IC50 | Rhodadyn-D10: DynI IC50 4.5 ± 0.8 µM; RME IC50 5.9 ± 1.0 µM. Rhodadyn-C10: RME IC50 7.0 ± 2.2 µM. | DynI GTPase assay; automated Texas Red-transferrin uptake (RME) in U2OS human osteosarcoma cells | No clathrin inhibition was detected at 100 µM; biochemical potency does not predict cellular activity for all Rhodadyn analogues. | Robertson et al., 2012 |
| Dynamin | Pyrimidyn (6/7) | Dynamin I/II GTPase + PH-domain phospholipid interaction | Biochemical IC50; cellular CME/SVE IC50 | Pyrimidyn-7: DynI IC50 1.1 ± 0.05 µM; DynII IC50 1.8 µM; CME IC50 12.1 ± 2.1 µM; SVE IC50 9.7 ± 2.5 µM. Pyrimidyn-6: DynI IC50 1.4 ± 0.2 µM; CME IC50 19.6 ± 3.5 µM; SVE IC50 10.7 ± 3.1 µM. | PS-stimulated GTPase assay (native sheep-brain DynI; full-length DynII); Tf-TxR/EGF-A488 uptake in non-neuronal cells (COS-7/U2OS/HER14); FM4-64 SVE assay in nerve terminals | Dual-action, GTP- and phospholipid-competitive mechanism; biochemical and cellular IC50 values should be reported separately. | McGeachie et al., 2013 |
| Off-target class | Wiskostatin | N-WASP/actin; off-target dynamin I GTPase and CME | Actin-assembly EC50; DynI GTPase IC50; cellular CME IC50 | PIP2-driven actin assembly EC50 ≈4 µM; purified N-WASP ≈60% inhibited at 10 µM (IC50 slightly <10 µM); DynI IC50 20.7 ± 1.2 µM; CME IC50 6.9 ± 0.3 µM | N-WASP: PIP2-triggered pyrene-actin assay in Xenopus egg extract (22 °C; first 6 min) and purified N-WASP/Arp2/3 assay. Off-target: helical DynI GTPase; U2OS fluorescent-transferrin CME (~30 min, 37 °C). | Polypharmacologic: N-WASP and dynamin/CME-active ranges overlap; not a selective clathrin inhibitor. | Peterson et al., 2004; Cossar et al., 2023 |
| Kinase/trafficking | Baricitinib | JAK1/2 established; NAK-related trafficking proposed | Clinical pharmacology / model-specific | Do not list a single CME potency | Coronavirus-related host-directed discussions | Clinical effects are not evidence of direct CCP inhibition | Richardson et al., 2020; Stebbing et al., 2020; Karim et al., 2022 |
| Endosomal pH | Bafilomycin A1 / Concanamycin A | V-ATPase / acidification | Working concentration | 10 nM-1 µM | Low-pH fusion/uncoating controls | Endosomal-acidification evidence, not CME proof | Yoshimori et al., 1991; Guinea and Carrasco, 1994 |
| Endosomal maturation | Apilimod / PIKfyve inhibitors | PIKfyve-dependent endosomal maturation | Working concentration / assay-dependent potency | 10 nM-1 µM | Filovirus/SARS-CoV-2-related entry screens | Downstream endosomal target, not direct CCP perturbation | Nelson et al., 2017; Kang et al., 2020; Riva et al., 2020 |
| Endosomal trafficking | EGA | Late-endosomal trafficking/escape | Working concentration | 10-25 µM | Toxin/virus trafficking assays | Not direct CME and not virus specific | Gillespie et al., 2013 |

**References**

Basagiannis, D., Zografou, S., Goula, E., Gkeka, D., Kolettas, E., and Christoforidis, S. (2021). Chemical inhibitors of dynamin exert differential effects in VEGF signaling. Cells 10, 997. doi: 10.3390/cells10050997.

Banbury, D. N., Oakley, J. D., Sessions, R. B., and Banting, G. (2003). Tyrphostin A23 inhibits internalization of the transferrin receptor by perturbing the interaction between tyrosine motifs and the medium chain subunit of the AP-2 adaptor complex. J. Biol. Chem. 278, 12022-12028. doi: 10.1074/jbc.M211966200.

Chen, Z., Mino, R. E., Mettlen, M., Michaely, P., Bhave, M., Reed, D. K., et al. (2020). Wbox2: a clathrin terminal domain-derived peptide inhibitor of clathrin-mediated endocytosis. J. Cell Biol. 219, e201908189. doi: 10.1083/jcb.201908189.

Cossar, P. J., Cardoso, D., Mathwin, D., Russell, C. C., Chiew, B., Hamilton, M. P., et al. (2023). Wiskostatin and other carbazole scaffolds as off target inhibitors of dynamin I GTPase activity and endocytosis. Eur. J. Med. Chem. 247, 115001. doi: 10.1016/j.ejmech.2022.115001.

Daniel, J. A., Chau, N., Abdel-Hamid, M. K., Hu, L., von Kleist, L., Whiting, A., et al. (2015). Phenothiazine-derived antipsychotic drugs inhibit dynamin and clathrin-mediated endocytosis. Traffic 16, 635-654. doi: 10.1111/tra.12272.

Dutta, D., Williamson, C. D., Cole, N. B., and Donaldson, J. G. (2012). Pitstop 2 is a potent inhibitor of clathrin-independent endocytosis. PLoS One 7, e45799. doi: 10.1371/journal.pone.0045799.

Elkin, S. R., Oswald, N. W., Reed, D. K., Mettlen, M., MacMillan, J. B., and Schmid, S. L. (2016). Ikarugamycin: a natural product inhibitor of clathrin-mediated endocytosis. Traffic 17, 1139-1149. doi: 10.1111/tra.12425.

Gillespie, E. J., Ho, C. L., Balaji, K., Clemens, D. L., Deng, G., Wang, Y. E., et al. (2013). Selective inhibitor of endosomal trafficking pathways exploited by multiple toxins and viruses. Proc. Natl. Acad. Sci. U.S.A. 110, E4904-E4912. doi: 10.1073/pnas.1302334110.

Gordon, C. P., Venn-Brown, B., Robertson, M. J., Young, K. A., Chau, N., Mariana, A., et al. (2013). Development of second-generation indole-based dynamin GTPase inhibitors. J. Med. Chem. 56, 46-59. doi: 10.1021/jm300844m.

Guinea, R., and Carrasco, L. (1994). Concanamycin A blocks influenza virus entry into cells under acidic conditions. FEBS Lett. 349, 327-330. doi: 10.1016/0014-5793(94)00695-4.

Hill, T. A., Gordon, C. P., McGeachie, A. B., Venn-Brown, B., Odell, L. R., Chau, N., et al. (2009). Inhibition of dynamin mediated endocytosis by the dynoles-synthesis and functional activity of a family of indoles. J. Med. Chem. 52, 3762-3773. doi: 10.1021/jm900036m.

Joshi, S., Perera, S., Gilbert, J., Smith, C. M., Mariana, A., Gordon, C. P., et al. (2010). The dynamin inhibitors MiTMAB and OcTMAB induce cytokinesis failure and inhibit cell proliferation in human cancer cells. Mol. Cancer Ther. 9, 1995-2006. doi: 10.1158/1535-7163.MCT-10-0161.

Kang, Y. L., Chou, Y. Y., Rothlauf, P. W., Liu, Z., Soh, T. K., Cureton, D., et al. (2020). Inhibition of PIKfyve kinase prevents infection by Zaire ebolavirus and SARS-CoV-2. Proc. Natl. Acad. Sci. U.S.A. 117, 20803-20813. doi: 10.1073/pnas.2007837117.

Karim, M., Saul, S., Ghita, L., Sahoo, M. K., Ye, C., Bhalla, N., et al. (2022). Numb-associated kinases are required for SARS-CoV-2 infection and are cellular targets for antiviral strategies. Antiviral Res. 204, 105367. doi: 10.1016/j.antiviral.2022.105367.

Macia, E., Ehrlich, M., Massol, R., Boucrot, E., Brunner, C., and Kirchhausen, T. (2006). Dynasore, a cell-permeable inhibitor of dynamin. Dev. Cell 10, 839-850. doi: 10.1016/j.devcel.2006.04.002.

McCluskey, A., Daniel, J. A., Hadzic, G., Chau, N., Clayton, E. L., Mariana, A., et al. (2013). Building a better dynasore: the Dyngo compounds potently inhibit dynamin and endocytosis. Traffic 14, 1272-1289. doi: 10.1111/tra.12119.

McGeachie, A. B., Odell, L. R., Quan, A., Daniel, J. A., Chau, N., Hill, T. A., et al. (2013). Pyrimidyn compounds: dual-action small molecule pyrimidine-based dynamin inhibitors. ACS Chem. Biol. 8, 1507-1518. doi: 10.1021/cb400137p.

Morgan, J. R., Prasad, K., Jin, S., Augustine, G. J., and Lafer, E. M. (2001). Uncoating of clathrin-coated vesicles in presynaptic terminals: roles for Hsc70 and auxilin. Neuron 32, 289-300. doi: 10.1016/S0896-6273(01)00467-6.

Nelson, E. A., Dyall, J., Hoenen, T., Barnes, A. B., Zhou, H., Liang, J. Y., et al. (2017). The phosphatidylinositol-3-phosphate 5-kinase inhibitor apilimod blocks filoviral entry and infection. PLoS Negl. Trop. Dis. 11, e0005540. doi: 10.1371/journal.pntd.0005540.

Odell, L. R., Howan, D., Gordon, C. P., Robertson, M. J., Chau, N., Mariana, A., et al. (2010). The pthaladyns: GTP competitive inhibitors of dynamin I and II GTPase derived from virtual screening. J. Med. Chem. 53, 5267-5280. doi: 10.1021/jm100442u.

Peterson, J. R., Bickford, L. C., Morgan, D., Kim, A. S., Ouerfelli, O., Kirschner, M. W., et al. (2004). Chemical inhibition of N-WASP by stabilization of a native autoinhibited conformation. Nat. Struct. Mol. Biol. 11, 747-755. doi: 10.1038/nsmb796.

Quan, A., McGeachie, A. B., Keating, D. J., van Dam, E. M., Rusak, J., Chau, N., et al. (2007). Myristyl trimethyl ammonium bromide and octadecyl trimethyl ammonium bromide are surface-active small molecule dynamin inhibitors that block endocytosis mediated by dynamin I or dynamin II. Mol. Pharmacol. 72, 1425-1439. doi: 10.1124/mol.107.034207.

Richardson, P., Griffin, I., Tucker, C., Smith, D., Oechsle, O., Phelan, A., et al. (2020). Baricitinib as potential treatment for 2019-nCoV acute respiratory disease. Lancet 395, e30-e31. doi: 10.1016/S0140-6736(20)30304-4.

Riva, L., Yuan, S., Yin, X., Martin-Sancho, L., Matsunaga, N., Pache, L., et al. (2020). Discovery of SARS-CoV-2 antiviral drugs through large-scale compound repurposing. Nature 586, 113-119. doi: 10.1038/s41586-020-2577-1.

Robertson, M. J., Hadzic, G., Ambrus, J., Pome, D. Y., Hyde, E., Whiting, A., et al. (2012). The Rhodadyns, a new class of small molecule inhibitors of dynamin GTPase activity. ACS Med. Chem. Lett. 3, 352-356. doi: 10.1021/ml200284s.

Sousa, R., and Lafer, E. M. (2015). The role of molecular chaperones in clathrin mediated vesicular trafficking. Front. Mol. Biosci. 2, 26. doi: 10.3389/fmolb.2015.00026.

Stebbing, J., Phelan, A., Griffin, I., Tucker, C., Oechsle, O., Smith, D., et al. (2020). COVID-19: combining antiviral and anti-inflammatory treatments. Lancet Infect. Dis. 20, 400-402. doi: 10.1016/S1473-3099(20)30132-8.

von Kleist, L., Stahlschmidt, W., Bulut, H., Gromova, K., Puchkov, D., Robertson, M. J., et al. (2011). Role of the clathrin terminal domain in regulating coated pit dynamics revealed by small molecule inhibition. Cell 146, 471-484. doi: 10.1016/j.cell.2011.06.025.

Wang, L. H., Rothberg, K. G., and Anderson, R. G. (1993). Mis-assembly of clathrin lattices on endosomes reveals a regulatory switch for coated pit formation. J. Cell Biol. 123, 1107-1117. doi: 10.1083/jcb.123.5.1107.

Yoshimori, T., Yamamoto, A., Moriyama, Y., Futai, M., and Tashiro, Y. (1991). Bafilomycin A1, a specific inhibitor of vacuolar-type H(+)-ATPase, inhibits acidification and protein degradation in lysosomes of cultured cells. J. Biol. Chem. 266, 17707-17712. doi: 10.1016/S0021-9258(19)47429-2.
